# Supplementary material for: Experiences of living with leprosy: A systematic review and qualitative evidence synthesis
Source: PLoS Negl Trop Dis. 2022 Oct 5;16(10):e0010761. doi: 10.1371/journal.pntd.0010761 (PMC9576094; doi:10.1371/journal.pntd.0010761)
Supplement: S1 Appendix — (DOCX) [file pntd.0010761.s001.docx]

## S1 Appendix

Overview of the search terms per database

### Table 1: Search strategy in PubMed

| **Search** | **Query** | **Results** |
| --- | --- | --- |
| **#6** | **#5 NOT ("Animals"[Mesh] NOT "Humans"[Mesh])** | **161** |
| **#5** | **#4 NOT (("Adolescent"[Mesh] OR "Child"[Mesh] OR "Infant"[Mesh] OR "adolescen*"[tiab] OR "child*" [tiab] OR "schoolchild*"[tiab] OR "infant*"[tiab] OR "girl*"[tiab] OR "boy"[tiab] OR "boys"[tiab] OR "teen*"[tiab] OR "youth*"[tiab] OR "pediatr*"[tiab] OR "paediatr*"[tiab] OR "pube*"[tiab]) NOT ("Adult"[Mesh] OR "adult*"[tiab] OR "man"[tiab] OR "men"[tiab] OR "woman"[tiab] OR "women"[tiab]))** | **164** |
| **#4** | **#1 AND #2 AND #3** | **175** |
| **#3** | **"Qualitative Research"[Mesh] OR "Focus Groups"[Mesh] OR "Interview" [Publication Type] OR "Interviews as Topic"[Mesh] OR "Narration"[Mesh] OR "Personal Narratives as Topic"[Mesh] OR "Observational Studies as Topic"[Mesh] OR "Observational Study"[Publication Type] OR "Tape Recording"[Mesh] OR "Grounded Theory"[Mesh] OR "thematic analys*"[tiab] OR "content analys*"[tiab] OR "focus group*"[tiab] OR "ethnograph*"[tiab] OR "ethnograf*"[tiab] OR "etnograf*"[tiab] OR "field stud*"[tiab] OR "phenomenolog*"[tiab] OR "narration*"[tiab] OR "narrative"[tiab] "case stud*"[tiab] OR "qualitative stud*"[tiab] OR "qualitative analys*"[tiab] OR "qualitative research*"[tiab] OR "qualitative method*"[tiab] OR "multimethodolog*"[tiab] OR "mixed method*"[tiab] OR "observation*"[tiab] OR "grounded theor*"[tiab] OR "audio recording*"[tiab] OR "tape recording*"[tiab] OR "audiotape*"[tiab] OR (("semi-structured"[tiab] OR "semistructured"[tiab] OR "unstructured"[tiab] OR "informal"[tiab] OR "in-depth"[tiab] OR "indepth"[tiab] OR "face-to-face"[tiab] OR "structured"[tiab] OR "guide*"[tiab]) AND ("interview*"[tiab] OR "discussion*"[tiab] OR "questionnaire*"[tiab]))** | **1,189,867** |
| **#2** | **"Quality of Life"[Mesh] OR "Severity of Illness Index"[Mesh] OR "Rehabilitation"[Mesh] OR "Health Status"[Mesh] OR "Models, Biopsychosocial"[Mesh] OR "quality of life"[tiab] OR "life qualit*"[tiab] OR "experience*"[tiab] OR "impact*"[tiab] OR "phenomenolog*"[tiab] OR "living"[tiab] OR "living qualit*"[tiab] OR "quality of living"[tiab] OR "activities of daily living"[tiab] OR "activity of daily living"[tiab] OR "activities of daily life"[tiab] OR "activity of daily life"[tiab] OR "daily living activit*"[tiab] OR "daily life activit*"[tiab] OR "adl"[tiab] OR "chronic limitation of activity"[tiab] OR "self care*"[tiab] OR "health status"[tiab] OR "level of health"[tiab] OR "health level*"[tiab] OR "qol"[tiab] OR "hrql"[tiab] OR "hrqol"[tiab] OR "biopsychosocial*"[tiab]** | **3,363,048** |
| **#1** | **"Leprosy"[Mesh] OR "Leprosy"[tiab] OR "Hansen s Dis*"[tiab] OR "Hansens Dis*"[tiab] OR "Hansen Dis*"[tiab]** | **26,342** |

### Table 2: Search strategy in Embase.com

| **Search** | **Query** | **Results** |
| --- | --- | --- |
| **#7** | #6 NOT ('conference abstract'/it OR 'conference paper'/it OR 'editorial'/it OR 'letter'/it) | **230** |
| **#6** | #5 NOT ([animals]/lim NOT [humans]/lim) | **283** |
| **#5** | #4 NOT (('juvenile'/exp OR 'embryo'/exp OR 'fetus'/exp OR (‘adolescen*’ OR ‘child*’ OR ‘schoolchild*’ OR ‘infant*’ OR ‘girl*’ OR ‘boy’ OR ‘boys’ OR ‘teen*’ OR ‘youth*’ OR ‘pediatr*’ OR ‘paediatr*’ OR ‘pube*’):ti,ab,kw) NOT ('adult'/exp OR (‘adult*’ OR ‘man’ OR ‘men’ OR ‘woman’ OR ‘women’):ti,ab,kw)) | **289** |
| **#4** | #1 AND #2 AND #3 | **303** |
| **#3** | 'qualitative research'/exp OR 'interview'/exp OR 'verbal communication'/exp OR 'observational study'/exp OR 'field study'/exp OR 'panel study'/exp OR 'recording'/exp OR 'grounded theory'/exp OR (‘thematic analys*’ OR ‘content analys*’ OR ‘focus group*’ OR ‘ethnograph*’ OR ‘ethnograf*’ OR ‘etnograf*’ OR ‘field stud*’ OR ‘phenomenolog*’ OR ‘narration*’ OR ‘narrative’ ‘case stud*’ OR ‘qualitative stud*’ OR ‘qualitative analys*’ OR ‘qualitative research*’ OR ‘qualitative method*’ OR ‘multimethodolog*’ OR ‘mixed method*’ OR ‘observation*’ OR ‘grounded theor*’ OR ‘audio recording*’ OR ‘tape recording*’ OR ‘audiotape*’ OR ((‘semi-structured’ OR ‘semistructured’ OR ‘unstructured’ OR ‘informal’ OR ‘in-depth’ OR ‘indepth’ OR ‘face-to-face’ OR ‘structured’ OR ‘guide*’) AND (‘interview*’ OR ‘discussion*’ OR ‘questionnaire*’))):ti,ab,kw | **2,220,754** |
| **#2** | 'quality of life'/exp OR 'severity of illness index'/exp OR 'rehabilitation'/exp OR 'health status'/exp OR 'biopsychosocial model'/exp OR (‘quality of life’ OR ‘life qualit*’ OR ‘experience*’ OR ‘impact*’ OR ‘phenomenolog*’ OR ‘living’ OR ‘living qualit*’ OR ‘quality of living’ OR ‘activities of daily living’ OR ‘activity of daily living’ OR ‘activities of daily life’ OR ‘activity of daily life’ OR ‘daily living activit*’ OR ‘daily life activit*’ OR ‘adl’ OR ‘chronic limitation of activity’ OR ‘self care*’ OR ‘health status’ OR ‘level of health’ OR ‘health level*’ OR ‘qol’ OR ‘hrql’ OR ‘hrqol’ OR ‘biopsychosocial*’):ti,ab,kw | **4,503,011** |
| **#1** | 'leprosy'/exp OR (‘Leprosy’ OR ‘Hansen s Dis*’ OR ‘Hansens Dis*’ OR ‘Hansen Dis*’):ti,ab,kw | **33,613** |

### Table 3: Search strategy in Clarivate Analytics/Web of Science Core Collection

| **Search** | **Query** | **Results** |
| --- | --- | --- |
| **#5** | #4 NOT TS=((“adolescen*” OR “child*” OR “schoolchild*” OR “infant*” OR “girl*” OR “boy” OR “boys” OR “teen*” OR “youth*” OR “pediatr*” OR “paediatr*” OR “pube*”) NOT (“adult*” OR “man” OR “men” OR “woman” OR “women”)) | **170** |
| **#4** | #1 AND #2 AND #3 | **180** |
| **#3** | TS=(“thematic analys*” OR “content analys*” OR “focus group*” OR “ethnograph*” OR “ethnograf*” OR “etnograf*” OR “field stud*” OR “phenomenolog*” OR “narration*” OR “narrative” “case stud*” OR “qualitative stud*” OR “qualitative analys*” OR “qualitative research*” OR “qualitative method*” OR “multimethodolog*” OR “mixed method*” OR “observation*” OR “grounded theor*” OR “audio recording*” OR “tape recording*” OR “audiotape*” OR ((“semi-structured” OR “semistructured” OR “unstructured” OR “informal” OR “in-depth” OR “indepth” OR “face-to-face” OR “structured” OR “guide*”) AND (“interview*” OR “discussion*” OR “questionnaire*”))) | **2,301,542** |
| **#2** | TS=(“quality of life” OR “life qualit*” OR “experience*” OR “impact*” OR “phenomenolog*” OR “living” OR “living qualit*” OR "quality of living" OR "activities of daily living" OR "activity of daily living" OR "activities of daily life" OR "activity of daily life" OR “daily living activit*” OR “daily life activit*” OR "adl" OR "chronic limitation of activity" OR “self care*” OR "health status" OR "level of health" OR “health level*” OR "qol" OR "hrql" OR "hrqol" OR “biopsychosocial*”) | **5,041,044** |
| **#1** | TS=("Leprosy" OR “Hansen s Dis*” OR “Hansens Dis*” OR “Hansen Dis*”) | **16,652** |

### Table 4: Search strategy in Cumulative Index to Nursing and Allied Health Literature (CINAHL).

| **Search** | **Query** | **Results** |
| --- | --- | --- |
| **#4** | #1 AND #2 AND #3 | **162** |
| **#3** | (MH "Qualitative Studies+") OR (MH "Focus Groups") OR (MH "Interviews+") OR (MH "Narratives+") OR (MH "Nonexperimental Studies+") OR (MH "Audiorecording") OR (MH "Videorecording+") OR (MH "Tapes+") OR (MH "Record Review") OR (MH "Grounded Theory") OR TI(“thematic analys*” OR “content analys*” OR “focus group*” OR “ethnograph*” OR “ethnograf*” OR “etnograf*” OR “field stud*” OR “phenomenolog*” OR “narration*” OR “narrative” “case stud*” OR “qualitative stud*” OR “qualitative analys*” OR “qualitative research*” OR “qualitative method*” OR “multimethodolog*” OR “mixed method*” OR “observation*” OR “grounded theor*” OR “audio recording*” OR “tape recording*” OR “audiotape*” OR ((“semi-structured” OR “semistructured” OR “unstructured” OR “informal” OR “in-depth” OR “indepth” OR “face-to-face” OR “structured” OR “guide*”) AND (“interview*” OR “discussion*” OR “questionnaire*”))) OR AB(“thematic analys*” OR “content analys*” OR “focus group*” OR “ethnograph*” OR “ethnograf*” OR “etnograf*” OR “field stud*” OR “phenomenolog*” OR “narration*” OR “narrative” “case stud*” OR “qualitative stud*” OR “qualitative analys*” OR “qualitative research*” OR “qualitative method*” OR “multimethodolog*” OR “mixed method*” OR “observation*” OR “grounded theor*” OR “audio recording*” OR “tape recording*” OR “audiotape*” OR ((“semi-structured” OR “semistructured” OR “unstructured” OR “informal” OR “in-depth” OR “indepth” OR “face-to-face” OR “structured” OR “guide*”) AND (“interview*” OR “discussion*” OR “questionnaire*”))) OR KW(“thematic analys*” OR “content analys*” OR “focus group*” OR “ethnograph*” OR “ethnograf*” OR “etnograf*” OR “field stud*” OR “phenomenolog*” OR “narration*” OR “narrative” “case stud*” OR “qualitative stud*” OR “qualitative analys*” OR “qualitative research*” OR “qualitative method*” OR “multimethodolog*” OR “mixed method*” OR “observation*” OR “grounded theor*” OR “audio recording*” OR “tape recording*” OR “audiotape*” OR ((“semi-structured” OR “semistructured” OR “unstructured” OR “informal” OR “in-depth” OR “indepth” OR “face-to-face” OR “structured” OR “guide*”) AND (“interview*” OR “discussion*” OR “questionnaire*”))) | **1,273,018** |
| **#2** | (MH "Quality of Life+") OR (MH "Severity of Illness Indices+") OR (MH "Rehabilitation+") OR (MH "Health Status+") OR (MH "Models, Biopsychosocial") OR TI(“quality of life” OR “life qualit*” OR “experience*” OR “impact*” OR “phenomenolog*” OR “living” OR “living qualit*” OR "quality of living" OR "activities of daily living" OR "activity of daily living" OR "activities of daily life" OR "activity of daily life" OR “daily living activit*” OR “daily life activit*” OR "adl" OR "chronic limitation of activity" OR “self care*” OR "health status" OR "level of health" OR “health level*” OR "qol" OR "hrql" OR "hrqol" OR “biopsychosocial*”) OR AB(“quality of life” OR “life qualit*” OR “experience*” OR “impact*” OR “phenomenolog*” OR “living” OR “living qualit*” OR "quality of living" OR "activities of daily living" OR "activity of daily living" OR "activities of daily life" OR "activity of daily life" OR “daily living activit*” OR “daily life activit*” OR "adl" OR "chronic limitation of activity" OR “self care*” OR "health status" OR "level of health" OR “health level*” OR "qol" OR "hrql" OR "hrqol" OR “biopsychosocial*”) OR KW(“quality of life” OR “life qualit*” OR “experience*” OR “impact*” OR “phenomenolog*” OR “living” OR “living qualit*” OR "quality of living" OR "activities of daily living" OR "activity of daily living" OR "activities of daily life" OR "activity of daily life" OR “daily living activit*” OR “daily life activit*” OR "adl" OR "chronic limitation of activity" OR “self care*” OR "health status" OR "level of health" OR “health level*” OR "qol" OR "hrql" OR "hrqol" OR “biopsychosocial*”) | **1,330,943** |
| **#1** | (MH "Leprosy") OR TI("Leprosy" OR “Hansen s Dis*” OR “Hansens Dis*” OR “Hansen Dis*”) OR AB("Leprosy" OR “Hansen s Dis*” OR “Hansens Dis*” OR “Hansen Dis*”) OR KW("Leprosy" OR “Hansen s Dis*” OR “Hansens Dis*” OR “Hansen Dis*”) | **1,914** |

Legend

### Table 1: Search strategy in PubMed

### Table 2: Search strategy in Embase.com

### Table 3: Search strategy in Clarivate Analytics/Web of Science Core Collection

### Table 4: Search strategy in Cumulative Index to Nursing and Allied Health Literature (CINAHL).
