# Supplementary material for: Experiences of living with leprosy: A systematic review and qualitative evidence synthesis
Source: PLoS Negl Trop Dis. 2022 Oct 5;16(10):e0010761. doi: 10.1371/journal.pntd.0010761 (PMC9576094; doi:10.1371/journal.pntd.0010761)
Supplement: S2 Appendix — (DOCX) [file pntd.0010761.s002.docx]

## S2 Appendix

**Excluded studies**

1. Barrett R. Self-mortification and the stigma of leprosy in northern India. 2005;19(2):30.

Reason for exclusion: Ineligible study design

1. Cross H, Sah AK. The experiences and attitudes of people affected by leprosy who voluntarily undertake leprosy services in Nepal. 2014;85(3):231.

Reason for exclusion: Ineligible phenomena of interest

1. Deepak S, Hansine PE, Braccini C. Self-care groups of leprosy-affected people in Mozambique. 2013;84(4):91.

Reason for exclusion: Ineligible phenomena of interest

1. Gonçalves CC, de Lacerda AF, Oliveira Santos HP. To see beyond an insensitive rash: the leprosy bearer has the word. 2009;11(128):45. Available from: https://search.ebscohost.com/login.aspx?direct=true&db=cin20&AN=105404963&site=ehost-live

Reason for exclusion: Article in Portuguese, no English translation

1. Gonçalves M, Rodrigues do Prado MA, Santana da Silva S, da Silva Santos K, de Araujo PN, Magali Fortuna C. Trabalho e hanseníase: as mulheres em suas dores, lutas e labutas. 2018;71:714. Available from: https://search.ebscohost.com/login.aspx?direct=true&db=cin20&AN=131246500&site=ehost-live

Reason for exclusion: Duplicate study

1. Govindharaj P, Srinivasan S, Darlong J. Social participation of persons affected by leprosy in an Endemic district, West Bengal, India. 2019;90(1):112. Available from: https://www.embase.com/search/results?subaction=viewrecord&id=L2002127472&from=export

Reason for exclusion: Ineligible study design

1. Heijnders ML. Experiencing leprosy: perceiving and coping with leprosy and its treatment. A qualitative study conducted in Nepal. 2004;75(4):37.

Reason for exclusion: Ineligible study design

1. John AS, Rao PSS, Das S. Assessment of needs and quality care issues of women with leprosy. 2010;81(1):40.

Reason for exclusion: Ineligible study design

1. Kim SL. Hansen’s Oral Life Histories and Healing. 2013;22(2):419.

Reason for exclusion: Article in Korean, No English Translation

1. Kumaresan JA, Maganu ET. Sociocultural Dimensions of Leprosy in North-Western Botswana. 1994;39(4):541.

Reason for exclusion: Ineligible study design

1. Luna IT, Beserra EP, Alves MDS, Pinheiro PN da C. Adhesion to Leprosy treatment: inherent difficulties of the patients. 2010;63(6):990. Available from: https://search.ebscohost.com/login.aspx?direct=true&db=cin20&AN=104865282&site=ehost-live

Reason for exclusion: Article in Portuguese, no English translation

1. Maia JA, Rêgo WR de S, Celestino JT, Celestino PT. Hansen’s disease: stigma and prejudice faced by inmates of Casa de Acolhida Souza Araujo in the city of Rio Branco. 2012;14(166):170. Available from: https://search.ebscohost.com/login.aspx?direct=true&db=cin20&AN=108186639&site=ehost-live

Reason for exclusion: Article in Portuguese, no English translation

1. Mellagi AG, Monteiro YN. [The religious imaginary of Hansen’s disease patients: a comparative study of former inmates of the asylums of São Paulo and current Hansen’s disease patients]. 2009;16(2):504.

Reason for exclusion: Article in Portuguese, no English translation

1. Nicholls PG, Wiens C, Smith WC. Delay in presentation in the context of local knowledge and attitude towards leprosy--the results of qualitative fieldwork in Paraguay. 2003;71(3):209.

Reason for exclusion: Ineligible study design

1. Ponomareff GL. Phenomenology of Delusions in a Case of Leprosy. 1965;121(12):1211.

Reason for exclusion: Ineligible phenomena of interest

1. Prochazka M, Timothy J, Pullan R, Kollie K, Rogers E, Wright A, et al. “Buruli ulcer and leprosy, they are intertwined”: Patient experiences of integrated case management of skin neglected tropical diseases in Liberia. 2020;14(2).

Reason for exclusion: Ineligible phenomena of interest

1. Proença Palmeira I, de Assunção Ferreira M. “O CORPO QUE EU FUI E O CORPO QUE EU SOU”: CONCEPÇÕES DE MULHERES COM ALTERAÇÕES CAUSADAS PELA HANSENÍASE. 2012;21(2):386. Available from: https://search.ebscohost.com/login.aspx?direct=true&db=cin20&AN=79452173&site=ehost-live

Reason for exclusion: Duplicate study

1. Rai SS, Irwanto, Peters RMH. Qualitative Exploration of Experiences and Consequences of Health-related Stigma among Indonesians with HIV, Leprosy, Schizophrenia and Diabetes. 2020;15(1):16.

Reason for exclusion: Ineligible phenomena of interest

1. Rai SS, Peters RMH, Syurina EV, Irwanto I, Naniche D, Zweekhorst MBM. Intersectionality and health-related stigma: insights from experiences of people living with stigmatized health conditions in Indonesia. 2020;19(1).

Reason for exclusion: Ineligible phenomena of interest

1. Sashida M, Nagata S, Murashima S, Haruna M. [Social rehabilitation experiences of people with a history of Hansen’s disease: interviews of readmitted residents in a sanatorium]. 2005;52(2):57.

Reason for exclusion: Article in Japanese, no English translation

1. Sousa Almeida AI, de Araujo Nogueira M, Janaú Feitosa EB, Carneiro Corrêa J, Sagica de Vasconcelos J, Ferreira de Sousa R, et al. MARCAS DO PASSADO: MEMÓRIAS E SENTIMENTOS DE (EX) PORTADORES DE HANSENÍASE RESIDENTES EM UM ANTIGO “LEPROSÁRIO.” 2018;9(4):17. Available from: https://search.ebscohost.com/login.aspx?direct=true&db=cin20&AN=134089897&site=ehost-live

Reason for exclusion: Article in Portuguese, no English translation

1. Staples J. Delineating disease: self-management of leprosy identities in South India. 2004;23(1):88.

Reason for exclusion: Ineligible study design

1. Staples J. Disguise, revelation and copyright: Disassembling the south Indian leper. 2003;9(2):315.

Reason for exclusion: Ineligible study design

1. Tanikawa T, Miyawaki H, Shinjyo H, Amano Y, Kondo M. The meaning of the experience of the everyday poverty of life for the oldest hansen’s disease survivors - Through the narratives of residents admitted around the second world war at a leprosarium in the Seto Inland sea. 2015;84(1):50. Available from: https://www.embase.com/search/results?subaction=viewrecord&id=L611890158&from=export http://dx.doi.org/10.5025/hansen.84.37

Reason for exclusion: Article in Japanese, no English translation

1. Van’t Noordende AT, Wubie Aycheh M, Tadesse T, Hagens T, Haverkort E, Schippers AP. A family-based intervention for prevention and self-management of disabilities due to leprosy, podoconiosis and lymphatic filariasis in Ethiopia: A proof of concept study. 2021;15(2).

Reason for exclusion: Ineligible study design

1. Wang CH. Mending a torn fish net: Parse’s theory-guided research on the lived experience of hope. 1997; Available from: https://search.ebscohost.com/login.aspx?direct=true&db=cin20&AN=109873805&site=ehost-live

Reason for exclusion: Ineligible phenomena of interest

1. White C. Leprosy and stigma in the context of international migration. 2011;82(2):54.

Reason for exclusion: Ineligible study design

1. Woodall P, Scollard D, Rajan L. Hansen Disease among Micronesian and Marshallese Persons Living in the United States. 2011;17(7):1208. Available from: https://search.ebscohost.com/login.aspx?direct=true&db=cin20&AN=104573979&site=ehost-live

Reason for exclusion: Ineligible phenomena of interest

1. Yang YK. [Life experiences of Korean patients with Hansen’s disease in Sorok Island Hospital]. 2014;44(6):48.

Reason for exclusion: Article in Korean, No English Translation

1. de Castro SMS, Watanabe HAW. The compulsory isolation of Hansen’s disease patients: Memories of the elderly. 2009;16(2):487. Available from: https://www.embase.com/search/results?subaction=viewrecord&id=L355033538&from=export

Reason for exclusion: Article in Portuguese, no English translation

1. de Oliveira MH, Gomes R, de Oliveira CM. [Leprosy and sexuality: living with a difference]. 1999;7(1):91.

Reason for exclusion: Duplicate study

1. de Oliveira MHP, Gomes R, de Oliveira CM. Leprosy and sexuality: living with the difference. 1999;7(1):91. Available from: https://search.ebscohost.com/login.aspx?direct=true&db=cin20&AN=107206747&site=ehost-live

Reason for exclusion: Article in Portuguese, no English translation

1. deSancha M, Jha K, Williams A. Health beliefs surrounding leprosy induced foot ulceration; an exploratory qualitative study from South Nepal. 2015;86(3):64.

Reason for exclusion: Ineligible phenomena of interest

Legend

**Excluded studies**
