# Supplementary material for: Experiences of living with leprosy: A systematic review and qualitative evidence synthesis
Source: PLoS Negl Trop Dis. 2022 Oct 5;16(10):e0010761. doi: 10.1371/journal.pntd.0010761 (PMC9576094; doi:10.1371/journal.pntd.0010761)
Supplement: S3 Appendix — (DOCX) [file pntd.0010761.s003.docx]

### S3 Appendix

**Included studies**

1. Abedi H, Javadi A, Naji S. An exploration of health, family and economic experiences of leprosy patients, Iran. Pak J Biol Sci. 2013;16: 927–32. doi:10.3923/pjbs.2013.927.932

2. Araújo de Souza I, Aparecido Ayres J, Meneguin S, Spagnolo RS. Hansen’s disease patients’ perception of self-care from the complexity perspective. Anna Nery School Journal of Nursing / Escola Anna Nery Revista de Enfermagem. 2014;18: 510–514. doi:10.5935/1414-8145.20140072

3. Ayres Jairo Aparecido;Paiva Bianca Sakamoto Ribeiro;Duarte Marli Teresinha Cassamassimo;Berti Heloisa Wey; Leprosy effects on patients’ daily lives: vulnerabili ty and solidarity. 2012;16: 62.

4. Carneiro da Silva RC, Ara£jo Vieira MC, Mistura C, Olinda de Souza Carvalho e Lira M, Sarmento SS. Estigma e preconceito: realidade de portadores de hansen¡ase em unidades prisionais. Revista de Pesquisa: Cuidado e Fundamental. 2014;6: 493–506. doi:10.9789/2175-5361.2014v6n2p493

5. Carvalho e Silva Sales J, Ribeiro de AraÃ^o^jo MP, Cavalcante Coelho M, LÃ^o^cia Evangelista de Sousa Luz V, AraÃ^o^jo da Silva TC, JosÃ© Guedes da Silva JÃ^o^nior F. SEXUALITY OF PEOPLE LIVING WITH LEPROSY: PERCEPTION AND REPERCUSSIONS. Journal of Nursing UFPE / Revista de Enfermagem UFPE. 2013;7: 460–466. doi:10.5205/reuol.3073-24791-1-LE.0702201318

6. Chen IJ, Cheng SP, Sheu SJ. The meaning of physical activity for older adults with leprosy: A life story inside the wall. Leprosy Review. 2017;88: 399–409.

7. Correia JC, Golay A, Lachat S, Singh SB, Manandhar V, Jha N, et al. “If you will counsel properly with love, they will listen”: A qualitative analysis of leprosy affected patients’ educational needs and caregiver perceptions in Nepal. PLoS One. 2019;14: e0210955. doi:10.1371/journal.pone.0210955

8. da Silva Duarte LMCP, Albino Simpson C, dos Santos Silva TM, de Lima Moura IB, Ramos Isoldi DM. SELF-CARE ACTIONS OF PEOPLE WITH LEPROSY. Journal of Nursing UFPE / Revista de Enfermagem UFPE. 2014;8: 2816–2822. doi:10.5205/reuol.6081-52328-1-SM.0808201432

9. da Silva Santos K, Magali Fortuna C, Fagundes Carvalho Gonçalves M, Matumoto S, Ribeiro Santana F, Marciano FM. Meaning of leprosy for people who have experienced treatment during the sulfonic and multidrug therapy periods. Revista Latino-Americana de Enfermagem (RLAE). 2015;23: 620–627. doi:10.1590/0104-1169.0323.2596

10. Da Silva MCD, Paz EPA. Experiences of people affected by leprosy in the health services: A hermeneutic approach. Leprosy Review. 2019;90: 172–182.

11. Dadun, Peters R, Lusli M, Miranda-Galarza B, van Brakel W, Zweekhorst M, et al. Exploring the Complexities of Leprosy-related Stigma and the Potential of a Socio-economic Intervention in a Public Health Context in Indonesia. Disability, CBR & Inclusive Development. 2016;27: 5–23. doi:10.5463/DCID.v27i3.551

12. Dako-Gyeke M, Asampong E, Oduro R. Stigmatisation and discrimination: Experiences of people affected by leprosy in Southern Ghana. Lepr Rev. 2017;88: 58–74.

13. Ebenso B, Ayuba M. “Money is the vehicle of interaction”: Insight into social integration of people affected by leprosy in Northern Nigeria. Leprosy Review. 2010;81: 99–110.

14. Goncalves M, Prado MAR do, Silva SS da, Santos K da S, Araujo PN de, Fortuna CM. Work and Leprosy: women in their pains, struggles and toils. Rev Bras Enferm. 2018;71: 660–667. doi:10.1590/0034-7167-2017-0598

15. Heijnders ML. Experiencing leprosy: perceiving and coping with leprosy and its treatment. A qualitative study conducted in Nepal. Lepr Rev. 2004;75: 327–37.

16. Jatimi A, Yusuf A, Andayani SRD. Leprosy Resilience with Disabilities Due to Illness: A Qualitative Study. Indonesian Nursing Journal of Education & Clinic (INJEC). 2020;5: 95–106. doi:10.24990/injec.v5i2.298

17. Jha K, Choudhary RK, Shrestha M, Sah A. An assessment of women’s empowerment in mixed self-help groups in dhanusha district of nepal. Leprosy Review. 2020;91: 155–172.

18. Jung HG, Yang YK. Disease experiences of female patients with Hansen’s disease residing in settlement in Korea. Int J Equity Health. 2020;19: 144. doi:10.1186/s12939-020-01264-7

19. Khanna D, de Wildt G, de Souza Duarte Filho LAM, Bajaj M, Lai JF, Gardiner E, et al. Improving treatment outcomes for leprosy in Pernambuco, Brazil: a qualitative study exploring the experiences and perceptions of retreatment patients and their carers. BMC Infect Dis. 2021;21: 282. doi:10.1186/s12879-021-05980-5

20. Sottie CA, Darkey J. Living with stigma: Voices from the Cured Lepers’ village in Ghana. Soc Work Health Care. 2019;58: 151–165. doi:10.1080/00981389.2018.1526842

21. Yusuf A, Aditya RS, Yunitasari E, Aziz AN, Solikhah FK. Experience of persons affected by leprosy in facing psychosocial problems: A qualitative method. Systematic Reviews in Pharmacy. 2020;11: 219–223. doi:10.31838/srp.2020.7.34

22. Van Netten WJ, Van Dorst MMAR, Waltz MM, Pandey BD, Aley D, Choudhary R, et al. Mental wellbeing among people affected by leprosy in the Terai region, Nepal. Leprosy Review. 2021;92: 59–74. doi:10.47276/lr.92.1.59

23. van Haaren MAC, Reyme M, Lawrence M, Menke J, Kaptein AA. Illness perceptions of leprosy-cured individuals in Surinam with residual disfigurements – “I am cured, but still I am ill.” Chronic Illness. 2017;13: 117–127. doi:10.1177/1742395316657398

24. van ’t Noordende AT, van Brakel WH, Banstola N, Dhakal KP. The Impact of Leprosy on Marital Relationships and Sexual Health among Married Women in Eastern Nepal. J Trop Med. 2016;2016: 4230235. doi:10.1155/2016/4230235

25. Try L. Gendered experiences: marriage and the stigma of leprosy. Asia Pacific Disability Rehabilitation Journal. 2006;17: 55–72.

26. Thompson L, Ioteba N, Chambers S. Leprosy in Kiribati: the lived experience. Leprosy Review. 2020;91: 353–366.

27. Susanto T, Dewi EI, Rahmawati I. The experiences of people affected by leprosy who participated in self-care groups in the community: A qualitative study in Indonesia. Leprosy Review. 2017;88: 543–553.

28. Steremberg Pires D’Azevedo S, Nunes de Freitas E, do Nascimento LO, dos Santos DCM, Delmondes do Nascimento R. PERCEPTION OF PATIENTS WITH LEPRA ABOUT THE SELF-CARE GROUPS. Journal of Nursing UFPE / Revista de Enfermagem UFPE. 2018;12: 1633–1639. doi:10.5205/1981-8963-v12i6a230855p1633-1639-2018

29. Silva CAB, Albuquerque VLM, Antunes MFR. Leprosy as a neglected disease and its stigma in the northeast of Brazil. Indian J Lepr. 2014;86: 53–59.

30. Sillo S, Lomax C, Wildt G, Da Silva Fonseca M. A temporal and sociocultural exploration of the stigma experiences of leprosy patients in Brazil. Leprosy review. 2016;87: 378–395.

31. Lima MCV, Barbosa FR, Santos D, Nascimento RDD, D’Azevedo SSP. Practices for self-care in Hansen’s disease: face, hands and feet. Rev Gaucha Enferm. 2018;39: e20180045. doi:10.1590/1983-1447.2018.20180045

32. Shyam-Sundar V, De Wildt G, Virmond MCL, Kyte D, Galan N, Prado, et al. A qualitative study exploring the perceived impact of race on leprosy-affected persons’ experiences of diagnosis and treatment of leprosy in southeast Brazil. Indian Journal of Leprosy. 2021;93: 1–13.

33. Shieh C, Wang HH, Lin CF. From contagious to chronic: A life course experience with leprosy in Taiwanese women. Leprosy Review. 2006;77: 99–113.

34. Schuller I, van Brakel WH, van der Vliet I, Beise K, Wardhani L, Silwana S, et al. The way women experience disabilities and especially disabilities related to leprosy in rural areas in south Sulawesi, Indonesia. Asia Pacific Disability Rehabilitation Journal. 2010;21: 60–70.

35. Ramasamy S, Govindharaj P, Kumar A, Panneerselvam S. Disclosure of Disease among Women affected by Leprosy: A Qualitative Study. Disability, CBR & Inclusive Development. 2020;31: 64–78. doi:10.47985/dcidj.393

36. Lusli M, Peters R, Bunders J, Irwanto I, Zweekhorst M. Development of a rights-based counselling practice and module to reduce leprosy-related stigma and empower people affected by leprosy in Cirebon District, Indonesia. Leprosy Review. 2017;88: 318–333.

37. Lusli M, Zweekhorst MBM, Miranda-Galarza B, Peters RMH, Cummings S, Seda FSSE, et al. Dealing with Stigma: Experiences of Persons Affected by Disabilities and Leprosy. Biomed Res Int. 2015;2015. doi:10.1155/2015/261329

38. Poestges H. Leprosy, the key to another kingdom. Lepr Rev. 2011;82: 155–67.

39. Peters RMH, Zweekhorst MBM, van Brakel WH, Bunders JFG, Irwanto. “People like me don’t make things like that”: Participatory video as a method for reducing leprosy-related stigma. Glob Public Health. 2016;11: 666–682. doi:10.1080/17441692.2016.1153122

40. Peters RMH, Dadun, Lusli M, Miranda-Galarza B, Van Brakel WH, Zweekhorst MBM, et al. The meaning of leprosy and everyday experiences: An exploration in Cirebon, Indonesia. Journal of Tropical Medicine. 2013. doi:10.1155/2013/507034

41. Pelizzari V, de Arruda GO, Marcon SS, Fernandes CAM. Perceptions of people with leprosy about disease and treatment. Rev Rene. 2016;17: 466–474. doi:10.15253/2175-6783.2016000400005

42. Nasir A, Yusuf A, Listiawan MY, Harianto S, Nuruddin, Huda N. Adaptive strategy of women’s leprosy in indonesia psychic experience of women with leprosy in living a community life. Systematic Reviews in Pharmacy. 2020;11: 306–312. doi:10.31838/srp.2020.10.51

43. Palmeira IP, Moura JN, Epifane SG, Ferreira AMR, Boulhosa MF. Hansen’s Disease Patients’ Perceptions on Their Altered Fundamental Human Needs: Indications for Self-Care. Revista De Pesquisa-Cuidado E Fundamental Online. 2020;12: 319–325. doi:10.9789/2175-5361.rpcfo.v12.7069

44. Palmeira IP, Ferreira MD. “the Body I Was and the Body I Am”: Conceptions of Women with Alterations Caused by Leprosy. Texto & Contexto Enfermagem. 2012;21: 379–386. doi:10.1590/S0104-07072012000200016

45. Nations MK, Lira GV, Catrib AM. Stigma, deforming metaphors and patients’ moral experience of multibacillary leprosy in Sobral, Ceará State, Brazil. Cad Saude Publica. 2009;25: 1215–24. doi:10.1590/s0102-311x2009000600004

46. Ebenso B.;Newell J.;Emmel N.;Adeyemi G.;Ola B.; Changing stigmatisation of leprosy: an exploratory, qualitative life course study in Western Nigeria. 2019;4.

47. Calcraft JH. The effects of the stigma of leprosy on the income generation of leprosy affected people in the Terai area of south east Nepal. Asia Pacific Disability Rehabilitation Journal. 2006;17: 73–89.

48. van ’t Noordende AT, Aycheh MW, Schippers A. The impact of leprosy, podoconiosis and lymphatic filariasis on family quality of life: A qualitative study in Northwest Ethiopia. PLoS Negl Trop Dis. 2020;14: e0008173. doi:10.1371/journal.pntd.0008173

49. Van’T Noordende AT, Lisam S, Ruthindartri P, Sadiq A, Singh V, Arifin M, et al. Leprosy perceptions and knowledge in endemic districts in india and indonesia: Differences and commonalities. PLoS Neglected Tropical Diseases. 2021;15: 1–19. doi:10.1371/journal.pntd.0009031

**Legend**

**Included studies**
