# Supplementary material for: Experiences of living with leprosy: A systematic review and qualitative evidence synthesis
Source: PLoS Negl Trop Dis. 2022 Oct 5;16(10):e0010761. doi: 10.1371/journal.pntd.0010761 (PMC9576094; doi:10.1371/journal.pntd.0010761)
Supplement: S4 Appendix — (DOCX) [file pntd.0010761.s004.docx]

S4 Appendix

**JBI checklist in JBI SUMARI** [1]

1. Congruity between the stated philosophical perspective and the research methodology

2. Congruity between the research methodology and the research question or objectives

3. Congruity between the research methodology and the methods used to collect data

4. Congruity between the research methodology and the representation and analysis of data

5. Congruity between the research methodology and the interpretation of results

6. Locating the researcher culturally or theoretically: Are their beliefs and values, and their potential influence on the study declared?

7. Influence of the researcher on the research, and vice-versa, is addressed.

8. Representation of participants and their voices: reports should provide illustrations from the data

9. Ethical approval by an appropriate body

10. Relationship of conclusions to analysis or interpretation of the data

**Reference**

1. Lockwood C, Munn Z, Porritt K. Qualitative research synthesis: methodological guidance for systematic reviewers utilizing meta-aggregation. JBI Evidence Implementation. 2015;13: 179–187. doi:10.1097/XEB.0000000000000062

**Legend**

JBI checklist in JBI SUMARI
