# Supplementary material for: Experiences of living with leprosy: A systematic review and qualitative evidence synthesis
Source: PLoS Negl Trop Dis. 2022 Oct 5;16(10):e0010761. doi: 10.1371/journal.pntd.0010761 (PMC9576094; doi:10.1371/journal.pntd.0010761)
Supplement: S5 Appendix — (DOCX) [file pntd.0010761.s005.docx]

### S5 Appendix

### Critical Appraisal Results

#### **Table of Critical appraisal results of eligible studies**

| **Citation** | **Q1** | **Q2** | **Q3** | **Q4** | **Q5** | **Q6** | **Q7** | **Q8** | **Q9** | **Q10** | **%** |
| --- | --- | --- | --- | --- | --- | --- | --- | --- | --- | --- | --- |
| Abedi H, Javadi A, Naji S. 2013. [1] | Y | Y | Y | Y | Y | N | N | Y | N | Y | 70 |
| Araújo de Souza I, Aparecido Ayres J, Meneguin S, Spagnolo RS. 2014. [2] | Y | Y | Y | U | Y | N | N | U | Y | Y | 60 |
| Ayres JA, Paiva BSR, Duarte MTC, Berti HW. 2012. [3] | Y | Y | Y | Y | Y | Y | Y | Y | Y | Y | 100 |
| Carneiro da Silva RC, Ara£jo Vieira MC, Mistura C, Olinda de Souza Carvalho e Lira M, Sarmento SS. 2014.[4] | Y | Y | Y | Y | Y | U | N | Y | Y | Y | 80 |
| Carvalho e Silva Sales J, Ribeiro de AraÃºjo MP, Cavalcante Coelho M, LÃºcia Evangelista de Sousa Luz V, AraÃºjo da Silva TC, JosÃ Guedes da Silva JÃºnior F. 2013.[5] | Y | Y | Y | Y | Y | Y | N | Y | Y | Y | 90 |
| Chen IJ, Cheng SP, Sheu SJ. 2017.[6] | Y | Y | Y | Y | Y | Y | N | U | Y | Y | 80 |
| Correia JC, Golay A, Lachat S, Singh SB, Manandhar V, Jha N, et al. 2019.[7] | Y | Y | Y | Y | Y | Y | N | Y | Y | Y | 90 |
| da Silva Duarte LMCP, Albino Simpson C, dos Santos Silva TM, de Lima Moura IB, Ramos Isoldi DM. 2014.[8] | Y | Y | Y | Y | Y | Y | N | Y | Y | Y | 90 |
| da Silva Santos K, Magali Fortuna C, Fagundes Carvalho Gonçalves M, Matumoto S, Ribeiro Santana F, Marciano FM. 2015.[9] | Y | Y | Y | Y | Y | Y | N | Y | Y | Y | 90 |
| Da Silva MCD, Paz EPA. 2019.[10] | Y | Y | Y | Y | Y | Y | N | Y | Y | Y | 90 |
| Dadun, Peters R, Lusli M, Miranda-Galarza B, van Brakel W, Zweekhorst M, et al. 2016.[11] | Y | Y | Y | Y | Y | Y | N | Y | Y | Y | 90 |
| Dako-Gyeke M, Asampong E, Oduro R. 2017.[12] | Y | Y | Y | Y | Y | Y | N | Y | Y | Y | 90 |
| Ebenso B, Ayuba M. 2010.[13] | Y | Y | Y | Y | Y | Y | N | Y | Y | Y | 90 |
| Gonçalves M, Prado M, Silva SSD, Santos KDS, Araujo PN, Fortuna CM. 2018.[14] | Y | Y | Y | Y | Y | Y | Y | Y | Y | Y | 100 |
| Heijnders ML. 2004.[15] | Y | Y | Y | Y | Y | Y | N | Y | Y | Y | 90 |
| Jatimi A, Yusuf A, Andayani SRD. 2020.[16] | Y | Y | Y | Y | Y | Y | N | Y | N | Y | 80 |
| Jha K, Choudhary RK, Shrestha M, Sah A. 2020.[17] | Y | Y | Y | Y | Y | Y | N | Y | Y | Y | 90 |
| Jung HG, Yang YK. 2020.[18] | Y | Y | Y | Y | Y | Y | N | Y | Y | Y | 90 |
| Khanna D, de Wildt G, de Souza Duarte Filho LAM, Bajaj M, Lai JF, Gardiner E, et al. 2021.[19] | Y | Y | Y | Y | Y | Y | Y | Y | Y | Y | 100 |
| Sottie CA, Darkey J. 2019.[20] | Y | Y | Y | Y | Y | U | N | Y | Y | Y | 90 |
| Yusuf A, Aditya RS, Yunitasari E, Aziz AN, Solikhah FK. 2020.[21] | Y | Y | Y | Y | Y | Y | N | Y | Y | Y | 90 |
| Van Netten WJ, Van Dorst MMAR, Waltz MM, Pandey BD, Aley D, Choudhary R, et al. 2021.[22] | Y | Y | Y | Y | Y | Y | N | Y | Y | Y | 90 |
| van Haaren MAC, Reyme M, Lawrence M, Menke J, Kaptein AA. 2017.[23] | Y | Y | Y | Y | Y | Y | N | Y | Y | Y | 90 |
| van ’t Noordende AT, van Brakel WH, Banstola N, Dhakal KP. 2016.[24] | Y | Y | Y | Y | Y | Y | Y | Y | N | Y | 90 |
| Try L. 2006.[25] | Y | Y | Y | Y | Y | U | N | Y | Y | Y | 80 |
| Thompson L, Iotebab N, Chambers S. 2020.[26] | Y | Y | Y | Y | Y | Y | N | Y | Y | Y | 90 |
| Susanto T, Dewi EI, Rahmawati I. 2017.[27] | Y | Y | Y | Y | Y | U | N | Y | Y | Y | 80 |
| Steremberg Pires D’Azevedo S, Nunes de Freitas E, do Nascimento LO, dos Santos DCM, Delmondes do Nascimento R. 2018.[28] | Y | Y | Y | Y | Y | Y | N | Y | U | Y | 80 |
| Silva CA, Albuquerque VL, Antunes MF. 2014.[29] | Y | Y | Y | Y | Y | Y | N | Y | Y | Y | 90 |
| Sillo S, Lomax C, De Wildt G, Fonseca MD, Galan NGD, Prado RBR. 2016.[30] | Y | Y | Y | Y | Y | Y | N | Y | Y | Y | 90 |
| Lima MCV, Barbosa FR, Santos D, Nascimento RDD, D’Azevedo SSP. 2018.[31] | Y | Y | Y | Y | Y | Y | N | Y | N | Y | 90 |
| Shyam-Sundar V, De Wildt G, Virmond MCL, Kyte D, Galan N, Prado, et al. 2021.[32] | Y | Y | Y | Y | Y | N | N | Y | Y | Y | 80 |
| Shieh C, Wang HH, Lin CF. 2006.[33] | Y | Y | Y | Y | Y | N | N | Y | N | Y | 80 |
| Schuller I, van Brakel WH, van der Vliet I, Beise K, Wardhani L, Silwana S, et al. 2010.[34] | Y | Y | Y | Y | Y | N | N | U | Y | Y | 80 |
| Ramasamy S, Govindharaj P, Kumar A, Panneerselvam S. 2020.[35] | Y | Y | Y | Y | Y | Y | N | Y | Y | Y | 90 |
| Lusli M, Peters R, Bunders J, Irwanto I, Zweekhorst M. 2017[36] | Y | Y | Y | Y | Y | Y | Y | Y | Y | Y | 100 |
| Lusli M, Zweekhorst MB, Miranda-Galarza B, Peters RM, Cummings S, Seda FS, et al. 2015.[37] | Y | Y | Y | Y | Y | Y | N | Y | Y | Y | 90 |
| Poestges H. 2011.[38] | Y | Y | Y | Y | Y | N | N | Y | Y | Y | 80 |
| Peters RM, Zweekhorst MB, van Brakel WH, Bunders JF, Irwanto. 2016.[39] | Y | Y | Y | Y | Y | U | N | Y | Y | Y | 80 |
| Peters RMH, Dadun, Lusli M, Miranda-Galarza B, Van Brakel WH, Zweekhorst MBM, et al. 2013.[40] | Y | Y | Y | Y | Y | N | N | U | Y | Y | 70 |
| Pelizzari V, de Arruda GO, Marcon SS, Fernandes CAM. 2016.[41] | Y | Y | Y | Y | Y | N | U | Y | N | Y | 70 |
| Nasir A, Yusuf A, Listiawan MY, Harianto S, Nuruddin, Huda N. 2020.[42] | Y | Y | Y | Y | Y | N | N | N | Y | Y | 70 |
| Palmeira IP, Moura JN, Epifane SG, Ferreira AMR, Boulhosa MF. 2020.[43] | Y | Y | Y | Y | Y | Y | N | Y | Y | Y | 90 |
| Palmeira IP, Ferreira MD. 2012[44] | Y | Y | Y | Y | Y | Y | N | Y | Y | Y | 90 |
| Nations MK, Lira GV, Catrib AM. 2009.[45] | Y | Y | Y | Y | Y | Y | N | Y | Y | Y | 90 |
| Ebenso B, Newell J, Emmel N, Adeyemi G, Ola B. 2019.[46] | Y | Y | Y | Y | Y | N | N | N | Y | Y | 70 |
| Calcraft JH. 2006.[47] | Y | Y | Y | Y | Y | N | N | Y | N | Y | 70 |
| van ’t Noordende AT, Aycheh MW, Schippers A. 2020.[48] | Y | Y | Y | Y | Y | N | N | Y | N | Y | 70 |
| Van’T Noordende AT, Lisam S, Ruthindartri P, Sadiq A, Singh V, Arifin M, et al. 2021.[49] | Y | Y | Y | Y | Y | Y | N | Y | N | Y | 80 |
| % | 100.0 | 100.0 | 100.0 | 97.95 | 100.0 | 65.3 | 10.2 | 87.75 | 79.59 | 100.0 | 82  84 |

References

1. Abedi H, Javadi A, Naji S. An exploration of health, family and economic experiences of leprosy patients, Iran. 2013;16(18):32.
2. Araújo de Souza I, Aparecido Ayres J, Meneguin S, Spagnolo RS. Hansen's disease patients' perception of self-care from the complexity perspective. 2014;18(3):514. Available from: https://search.ebscohost.com/login.aspx?direct=true&db=cin20&AN=97766381&site=ehost-live
3. Ayres JA, Paiva BSR, Duarte MTC, Berti HW. Leprosy effects on patients' daily lives: vulnerabili ty and solidarity. 2012;16(1):62. Available from: https://search.ebscohost.com/login.aspx?direct=true&db=cin20&AN=108100795&site=ehost-live
4. Calcraft JH. The effects of the stigma of leprosy on the income generation of leprosy affected people in the Terai area of south east Nepal. 2006;17(2):89. Available from: https://search.ebscohost.com/login.aspx?direct=true&db=cin20&AN=106104567&site=ehost-live
5. Carneiro da Silva RC, Ara£jo Vieira MC, Mistura C, Olinda de Souza Carvalho e Lira M, Sarmento SS. Estigma e preconceito: realidade de portadores de hansen¡ase em unidades prisionais. 2014;6(2):506. Available from: https://search.ebscohost.com/login.aspx?direct=true&db=cin20&AN=95411844&site=ehost-live
6. Carvalho e Silva Sales J, Ribeiro de AraÃºjo MP, Cavalcante Coelho M, LÃºcia Evangelista de Sousa Luz V, AraÃºjo da Silva TC, JosÃ© Guedes da Silva JÃºnior F. SEXUALITY OF PEOPLE LIVING WITH LEPROSY: PERCEPTION AND REPERCUSSIONS. 2013;7(2):466. Available from: https://search.ebscohost.com/login.aspx?direct=true&db=cin20&AN=104262622&site=ehost-live
7. Chen IJ, Cheng SP, Sheu SJ. The meaning of physical activity for older adults with leprosy: A life story inside the wall. 2017;88(3):409.
8. Correia JC, Golay A, Lachat S, Singh SB, Manandhar V, Jha N, et al. "If you will counsel properly with love, they will listen": A qualitative analysis of leprosy affected patients' educational needs and caregiver perceptions in Nepal. 2019;14(2).
9. Da Silva MCD, Paz EPA. Experiences of people affected by leprosy in the health services: A hermeneutic approach. 2019;90(2):182. Available from: https://www.embase.com/search/results?subaction=viewrecord&id=L2002356874&from=export
10. Dadun, Peters R, Lusli M, Miranda-Galarza B, van Brakel W, Zweekhorst M, et al. Exploring the Complexities of Leprosy-related Stigma and the Potential of a Socio-economic Intervention in a Public Health Context in Indonesia. 2016;27(3):23. Available from: https://search.ebscohost.com/login.aspx?direct=true&db=cin20&AN=126291194&site=ehost-live
11. Dako-Gyeke M, Asampong E, Oduro R. Stigmatisation and discrimination: Experiences of people affected by leprosy in Southern Ghana. 2017;88(1):74.
12. Ebenso B, Ayuba M. "Money is the vehicle of interaction": Insight into social integration of people affected by leprosy in Northern Nigeria. 2010;81(2):110. Available from: https://www.embase.com/search/results?subaction=viewrecord&id=L360082239&from=export http://www.leprahealthinaction.org/lr/June10/Lep99-110.pdf
13. Ebenso B, Newell J, Emmel N, Adeyemi G, Ola B. Changing stigmatisation of leprosy: an exploratory, qualitative life course study in Western Nigeria. 2019;4(2).
14. Gonçalves M, Prado M, Silva SSD, Santos KDS, Araujo PN, Fortuna CM. Work and Leprosy: women in their pains, struggles and toils. 2018;71(suppl 1):667.
15. Heijnders ML. The dynamics of stigma in leprosy. 2004;72(4):47.
16. Jatimi A, Yusuf A, Andayani SRD. Leprosy Resilience with Disabilities Due to Illness: A Qualitative Study. 2020;5(2):106. Available from: https://search.ebscohost.com/login.aspx?direct=true&db=cin20&AN=145620237&site=ehost-live
17. Jha K, Choudhary RK, Shrestha M, Sah A. An assessment of women's empowerment in mixed self-help groups in dhanusha district of nepal. 2020;91(2):172. Available from: https://www.embase.com/search/results?subaction=viewrecord&id=L2005039970&from=export
18. Jung HG, Yang YK. Disease experiences of female patients with Hansen's disease residing in settlement in Korea. 2020;19(1).
19. Khanna D, de Wildt G, de Souza Duarte Filho LAM, Bajaj M, Lai JF, Gardiner E, et al. Improving treatment outcomes for leprosy in Pernambuco, Brazil: a qualitative study exploring the experiences and perceptions of retreatment patients and their carers. 2021;21(1).
20. Lima MCV, Barbosa FR, Santos D, Nascimento RDD, D’Azevedo SSP. Practices for self-care in Hansen's disease: face, hands and feet. 2018;39.
21. Lusli M, Peters R, Bunders J, Irwanto I, Zweekhorst M. Development of a rights-based counselling practice and module to reduce leprosy-related stigma and empower people affected by leprosy in Cirebon District, Indonesia. 2017;88(3):333.
22. Lusli M, Zweekhorst MB, Miranda-Galarza B, Peters RM, Cummings S, Seda FS, et al. Dealing with stigma: experiences of persons affected by disabilities and leprosy. 2015;2015.
23. Nasir A, Yusuf A, Listiawan MY, Harianto S, Nuruddin, Huda N. Adaptive strategy of women's leprosy in indonesia psychic experience of women with leprosy in living a community life. 2020;11(10):312. Available from: https://www.embase.com/search/results?subaction=viewrecord&id=L2010035386&from=export http://dx.doi.org/10.31838/srp.2020.10.51
24. Nations MK, Lira GV, Catrib AM. Stigma, deforming metaphors and patients' moral experience of multibacillary leprosy in Sobral, Ceará State, Brazil. 2009;25(6):24.
25. Palmeira IP, Ferreira MD. "the Body I Was and the Body I Am": Conceptions of Women with Alterations Caused by Leprosy. 2012;21(2):386.
26. Palmeira IP, Moura JN, Epifane SG, Ferreira AMR, Boulhosa MF. Hansen's Disease Patients' Perceptions on Their Altered Fundamental Human Needs: Indications for Self-Care. 2020;12:325.
27. Pelizzari V, de Arruda GO, Marcon SS, Fernandes CAM. Perceptions of people with leprosy about disease and treatment. 2016;17(4):474.
28. Peters RM, Zweekhorst MB, van Brakel WH, Bunders JF, Irwanto. "People like me don" t make things like that': Participatory video as a method for reducing leprosy-related stigma. 2016;11(5–6):82.
29. Peters RMH, Dadun, Lusli M, Miranda-Galarza B, Van Brakel WH, Zweekhorst MBM, et al. The meaning of leprosy and everyday experiences: An exploration in Cirebon, Indonesia. 2013; Available from: https://www.embase.com/search/results?subaction=viewrecord&id=L368783519&from=export http://dx.doi.org/10.1155/2013/507034
30. Poestges H. Leprosy, the key to another kingdom. 2011;82(2):67.
31. Ramasamy S, Govindharaj P, Kumar A, Panneerselvam S. Disclosure of Disease among Women affected by Leprosy: A Qualitative Study. 2020;31(3):78. Available from: https://search.ebscohost.com/login.aspx?direct=true&db=cin20&AN=147795582&site=ehost-live
32. Schuller I, van Brakel WH, van der Vliet I, Beise K, Wardhani L, Silwana S, et al. The way women experience disabilities and especially disabilities related to leprosy in rural areas in south Sulawesi, Indonesia. 2010;21(1):70. Available from: https://search.ebscohost.com/login.aspx?direct=true&db=cin20&AN=105140920&site=ehost-live
33. Shieh C, Wang HH, Lin CF. From contagious to chronic: A life course experience with leprosy in Taiwanese women. 2006;77(2):113. Available from: https://www.embase.com/search/results?subaction=viewrecord&id=L44076488&from=export http://www.lepra.org.uk/lr/June06/Lep99-113.pdf
34. Shyam-Sundar V, De Wildt G, Virmond MCL, Kyte D, Galan N, Prado, et al. A qualitative study exploring the perceived impact of race on leprosy-affected persons' experiences of diagnosis and treatment of leprosy in southeast Brazil. 2021;93(1):13. Available from: https://www.embase.com/search/results?subaction=viewrecord&id=L2006896936&from=export
35. Sillo S, Lomax C, De Wildt G, Fonseca MD, Galan NGD, Prado RBR. A temporal and sociocultural exploration of the stigma experiences of leprosy patients in Brazil. 2016;87(3):395.
36. Silva CA, Albuquerque VL, Antunes MF. Leprosy as a neglected disease and its stigma in the northeast of Brazil. 2014;86(2):9.
37. Sottie CA, Darkey J. Living with stigma: Voices from the Cured Lepers' village in Ghana. 2019;58(2):165.
38. Steremberg Pires D’Azevedo S, Nunes de Freitas E, do Nascimento LO, dos Santos DCM, Delmondes do Nascimento R. PERCEPTION OF PATIENTS WITH LEPRA ABOUT THE SELF-CARE GROUPS. 2018;12(6):1639. Available from: https://search.ebscohost.com/login.aspx?direct=true&db=cin20&AN=130259134&site=ehost-live
39. Susanto T, Dewi EI, Rahmawati I. The experiences of people affected by leprosy who participated in self-care groups in the community: A qualitative study in Indonesia. 2017;88(4):553.
40. Thompson L, Iotebab N, Chambers S. Leprosy in kiribati: The lived experience. 2020;91(4):366. Available from: https://www.embase.com/search/results?subaction=viewrecord&id=L2005896536&from=export http://dx.doi.org/10.47276/lr.91.4.353
41. Try L. Gendered experiences: marriage and the stigma of leprosy. 2006;17(2):72. Available from: https://search.ebscohost.com/login.aspx?direct=true&db=cin20&AN=106104564&site=ehost-live
42. Van Netten WJ, Van Dorst MMAR, Waltz MM, Pandey BD, Aley D, Choudhary R, et al. Mental wellbeing among people affected by leprosy in the Terai region, Nepal. 2021;92(1):74. Available from: https://www.embase.com/search/results?subaction=viewrecord&id=L2006871439&from=export http://dx.doi.org/10.47276/lr.92.1.59
43. Van'T Noordende AT, Lisam S, Ruthindartri P, Sadiq A, Singh V, Arifin M, et al. Leprosy perceptions and knowledge in endemic districts in india and indonesia: Differences and commonalities. 2021;15(1):19. Available from: https://www.embase.com/search/results?subaction=viewrecord&id=L2005928049&from=export http://dx.doi.org/10.1371/journal.pntd.0009031
44. Yusuf A, Aditya RS, Yunitasari E, Aziz AN, Solikhah FK. Experience of persons affected by leprosy in facing psychosocial problems: A qualitative method. 2020;11(7):223. Available from: https://www.embase.com/search/results?subaction=viewrecord&id=L2007694874&from=export http://dx.doi.org/10.31838/srp.2020.7.34
45. da Silva Duarte LMCP, Albino Simpson C, dos Santos Silva TM, de Lima Moura IB, Ramos Isoldi DM. SELF-CARE ACTIONS OF PEOPLE WITH LEPROSY. 2014;8(8):2822. Available from: https://search.ebscohost.com/login.aspx?direct=true&db=cin20&AN=103983823&site=ehost-live
46. da Silva Santos K, Magali Fortuna C, Fagundes Carvalho Gonçalves M, Matumoto S, Ribeiro Santana F, Marciano FM. Meaning of leprosy for people who have experienced treatment during the sulfonic and multi-drug therapy periods. 2015;23(4):627. Available from: https://search.ebscohost.com/login.aspx?direct=true&db=cin20&AN=110372864&site=ehost-live
47. van Haaren MAC, Reyme M, Lawrence M, Menke J, Kaptein AA. Illness perceptions of leprosy-cured individuals in Surinam with residual disfigurements – "I am cured, but still I am ill." 2017;13(2):127. Available from: https://search.ebscohost.com/login.aspx?direct=true&db=cin20&AN=123343660&site=ehost-live
48. van ’t Noordende AT, Aycheh MW, Schippers A. The impact of leprosy, podoconiosis and lymphatic filariasis on family quality of life: A qualitative study in Northwest Ethiopia. 2020;14(3).
49. van ’t Noordende AT, van Brakel WH, Banstola N, Dhakal KP. The Impact of Leprosy on Marital Relationships and Sexual Health among Married Women in Eastern Nepal. 2016;2016.

**Legend**

#### Table of Critical appraisal results of eligible studies
