# Supplementary material for: Experiences of living with leprosy: A systematic review and qualitative evidence synthesis
Source: PLoS Negl Trop Dis. 2022 Oct 5;16(10):e0010761. doi: 10.1371/journal.pntd.0010761 (PMC9576094; doi:10.1371/journal.pntd.0010761)
Supplement: S6 Appendix — (DOCX) [file pntd.0010761.s006.docx]

S6 Appendix

**List of Study Findings with Illustrations (Key: C=Credible, N=Unsupported, U=Unequivocal)**

| Study: An exploration of health, family and economic experiences of leprosy patients, Iran 2013 Abedi, H; Javadi, A.; Naji, S [1] | |
| --- | --- |
| Finding | Health Experiences (C) |
| Illustration | My parents were both suffering from leprosy, I also got the disease, we generally were a few families who had to live far from the village. Lack of health facilities, poverty as well as neglecting me by my parents led my disease symptoms evident, and during a few years, my face deformed to current shape (destroyed nose cartilage, flat swelling face, losing eyebrows). (p4)  After the death of my parents, the government had asked the people who lived far from the area, went to Tabriz Leprosy Village and stayed there until five years ago. During this time I never think about marriage and even I didn't want to think of this issue as well, because I knew no one accept me as a husband. (P6)  Now, I have no sense of smell, no job or income, just people help me for sake of God. I wish God kills me as soon as possible to get me from this painful life. For many years I have been alone, no one sit next to me and talk to me for even a few moments. (P9) |
| Finding | Family experiences (C) |
| Illustration | People should know that we are also human beings and we have the right to live, we have our case like a harvest that is on fire and others that wood is. never my family understand me, they never support me, they left me alone with a huge problems. |
| Finding | Economic experiences (C) |
| Illustration | no one accepts me as worker and I had never job, no income, I am disappoint |
| Study: Hansen's disease patients' perception of self-care from the complexity perspective 2014 Araújo de Souza, Ioná; Aparecido Ayres, Jairo; Meneguin, Silmara; Spagnolo, Regina Stella [2] | |
| Finding | To be a person with Hansen's Disease (C) |
| Illustration | We have to accept it as if it were part of life because there is no other option. I have suffered a lot with it. It is awful to have leprosy. I was shocked to see the huge patches on my body and did not know what they were. I am always embarrassed and afraid of what people are going to think and many people end up staying away. People used to ask what I had and I used to cope with the asking as well as I could until, one day, my wife said it was all very embarrassing.  My legs went numb, I couldn't walk anymore and they still haven't gone back to normal. I don't think they will ever be the same again but, if they do go back to being what they were, what are things going to be like?  When I am on the streets I don't go near people anymore, you see, people look and they are afraid of catching it... |
| Finding | Self-care (C) |
| Illustration | I don't know much about that self-care stuff, I look after my body well and I shower. I think it has to do with knowing how to look after yourself well and live according to the instruction we are given. One has to protect well the skin and feet to not get hurt. I follow the instructions very carefully and I think that is what self-care means so one doesn't make things worse.  I have taken the medication because of the skin lesions and they are disappearing, I think that, if I had not looked after myself, I wouldn't have gotten better.  The thing I cannot do is rest because I am not a retired person yet.  Although I do a lot, it is still not enough, I have to take greater care but I find it difficult.  But I really don't care that much about that stuff, I don't really take care, I have to work out in the field, cook lunch and dinner, life carries on as usual... |
| Finding | Drug therapy (C) |
| Illustration | I don't know the difference. I take a little red one and a little white one and I know they are for Hansen's Disease but I don't know what they are for, I know they eliminate the problem. It is a drug that kills the microbes in the body, the bad guys. When I take a corticoid it is to stop the pain in my nerves and then the bumps disappear from my body. Thalidomide was the drug I most took which is for inhibiting the problem in the nodes... |
| Finding | Lifestyle of a person with Hansen's Disease (C) |
| Illustration | The truth is nothing has changed. I started the treatment and it is one extra thing I need to remember to do every day.  I used to do things that I cannot do anymore, I cannot work, I don't have any strength in my arms anymore nor in my legs like I used to have.  I had to start water aerobics lessons, I had never practiced any walking exercises nor stretching. My legs, arms, toes, fingers and joints in my arms hurt for a while and then it stops.  At first I was a bit depressed. My wife almost left me, she dumped a lot of stuff on me. It was as if she put me down and stepped all over me... |
| Study: Leprosy effects on patients' daily lives: vulnerability and solidarity 2012 Ayres, Jairo Aparecido; Paiva, Bianca Sakamoto Ribeiro; Duarte, Marli Teresinha Cassamassimo; Berti, Heloisa Wey [3] | |
| Finding | The challenges of living with symptoms and signs of leprosy (C) |
| Illustration | It's terrible, you get hurt and you don't feel anything, if you burn you don't feel it, when you see it it's already burned, the skin is dead.  It brought me a stain on my body, a lot of pain, so I had to operate. It made me very nervous, the worst thing was that I didn't think it would heal.  It was very difficult (crying) maybe you feel the way to death (crying) is more difficult to understand, until you overcome [...].  At the time, we were afraid of people picking up and moving away from us, especially the person we live with, who lives together, the family [...]. We get a little nervous, afraid to pass on another one.  I just stay at home, I don't go for walks, I just stay with my family... And it's not that I'm excluded from things, I'm excluded in my head; if you become psychologically weak you are excluded, you exclude yourself actually. |
| Finding | Relational life and leprosy (C) |
| Illustration | There are people who ask and I lie, I say that this is rheumatism (laughs). I even avoid talking because there are people who, if we talk, move away.  This is not something that is contagious, it was contagious in the beginning of the world, now there is treatment.  They say there is no danger of transmitting it to another with the treatment. So, the person is prejudiced, and that hurts a little, we feel, but we have to face it.  For other people who don't really know the disease just by talking [...]. So we use the term that is an inflammation, a very accentuated, very strong neuritis, so I have not spoken openly its old name or that it is leprosy, because people do not really understand what the disease is.  I only talk to my sisters and my brothers don't. [...] It was very surprising and we are living until now. I also talk to my husband and family about the problem of the disease. With outsiders, no, because of prejudice. |
| Finding | Daily life (C) |
| Illustration | There are many problems for us due to the disease.  I don't have a problem because I avoid getting hurt. It's like not being in the presence of God, we talk and nobody listens. I can't think fast. |
| Study: Estigma e preconceito: realidade de portadores de hansen¡ase em unidades prisionais 2014 Carneiro da Silva, Raquel Caroline; Ara£jo Vieira, Michelle Christini; Mistura, Claudelí; Olinda de Souza Carvalho e Lira, Margaret; Sarmento, Sued Sheila [4] | |
| Finding | Leprosy from the perspective of patients and family relations (C) |
| Illustration | I see myself as a normal person, the same way, without any prejudice. If you are doing the treatment has no prejudice, no catch as more. (Hefesto)  I would not be afraid to come in person, would not be prejudiced in any way. (Afrodite)  I see a person with leprosy, a person in need of help, why has no one dies to "starve". (Atena)  I "hunted" a way to stay as far away as possible from my little children, not to have contact to get them, you know. So I was far from them a way of not passing disease to them and to the staff inside. (Hefesto) |
| Finding | Stigma of leprosy, the impact of diagnosis and its feeling (C) |
| Illustration | The staff there from the street moved, were farthest from me, commented that they were afraid to touch and grab. (Hefesto)  I feel sad to have this disease, just wanted to take care. I'm ashamed because of stains, afraid of anyone knowing the disease, the spots get afraid of losing my family because of it. (Dionísio)  I felt anxious without knowing it, thought it was incurable. Before I was cheerful, happy. After these things, I was sadder more anxious, I was very skinny and worried. (Hefesto)  I was very nervous, crying, desperate. It was very bad, it was very sad, Ave Maria, I thought the world would end for me and that would not exist for most world I live, I was just nervous, I entered the same trauma. (Afrodite) |
| Finding | Denying leprosy and using divine faith to face the disease. (C) |
| Illustration | Nobody knows nothing, no one comes to visit me only my wife, and she does not know, I had not the courage to tell, I am ashamed and afraid to tell, for her not to worry. (Dionísio)  Always beats a weakness in me, then I go to the corner to ask God to give me strength. I pray to God that he has cured me and do not let more I go through this situation. (Atena) |
| Study:: PERCEPTION AND REPERCUSSIONS 2013 [5] | |
| Finding | Sexuality as a synonym for sex in the view of the leprosy patient (C) |
| Illustration | I don ́t know what else to say because of this I have already stopped my life for some time now [ ... ] my life today that I'm speaking about, you have no [ ... ]I don ́t even think [ ... ] I don ́t even stop to think about sex. (D1)  [ ... ] It is very "brutal", only that I don ́t do it without [...] I satisfy myself, I cum. In my sex life still, I am not "frustrated" because I ́m not what I was before, but it not over yet. (D2)  [ ... ] for men I think is very important, that if he does not practice he has no health; So I think that is a very important thing for the men ́s life as for the woman's life. (D3)  It ́s normal because human life was to be this way, among couples. [ ... ] because God left sexuality within marriage which is normal to build a family, but outside of marriage is not certain. (D4) |
| Finding | Repercussions of leprosy in the sexuality of people with the disease (C) |
| Illustration | [...] It gives me some cramps and I feel weakness in the nerves, I feel badly with this ordeal, and when the weather is hot I feel much more [ ... ] I think that this ordeal of sexuality comes from the nerve and the disease is more in the nerve, and there also the person which is concerned with this disease throughout life [ ... ] that is why I wanted to be good. (D7)  [...] A difficult time, because we has a problem with this, we always feel constant pain and that is not good. (D8)  ....I think it does all this with us, transforms the body of people, so that we are left with shame, our bodies change. |
| Study: The meaning of physical activity for older adults with leprosy: A life story inside the wall 2017 Chen, I. J; Cheng, S. P.; Sheu, S. J [6] | |
| Finding | Physical activity is a natural component of life (C) |
| Illustration | natural tendency: Nothing is special. I go out after I get up in the morning. Our body feels strange if we don’t exercise.  part of everyday routine: This is something we must do. It’s a routine. We do not get to choose. We have to do it every day. |
| Finding | Physical activity is beneficial to one's body and mind (C) |
| Illustration | health benefits: It’s good for our body. It’s good for our health. I do not often feel pain in my body. It also makes me look better. My body will become more flexible instead of more rigid.  brings me joy, peace and hope: I am doing rehabilitation. I feel peaceful. I feel relaxed and enthusiastic! empowers me: I can still walk and I do not want to die so soon. So I keep an active life-style. If possible, I would try my best to perform physical activity, and I can still care for myself. Do not over-exercise. Do it slowly. Do not ask yourself to achieve inappropriate goals. |
| Finding | Difficulty with physical activity is a degrading reminder of leprosy (C) |
| Illustration | brings out bitterness and frustrations from my past: Look at me. I cannot even walk well, let alone run or jump. If I were not amputated, I could walk the whole day.  exposes my inferiority: I am afraid of how others view me. How can we exercise with people without leprosy? How could we dare to exercise with people outside the sanatorium? |
| Finding | Physical activity is the acceptance of one's life circumstances (C) |
| Illustration | reminder of one's co-existence with leprosy: I do not move that well, so I do what I can do.  provides a field for one's cultivation: Sometimes, I think it’s better to see my life from another perspective, so that I do not feel depressed. Because my hands do not move anymore, I quit cigarettes and alcohol. It’s better! |
| Study: "If you will counsel properly with love, they will listen": A qualitative analysis of leprosy affected patients' educational needs and caregiver perceptions in Nepal 2019 [7] | |
| Finding | Socio-professional dimension (C) |
| Illustration | “I have difficulties holding items. My wounds make it difficult to plough land.” (lalP2) |
| Finding | Biomedical dimension (C) |
| Illustration | I did not know it is serious. I got something like blisters from burns from fire. I did not give much attention to the wounds. My friend recommended me to come here. Now I have wounds as well as deformities. (LalP1)  For 2–3 months I could not work, as I was weak. I could not go to get water (2 hours walk) and I did less household works. Also, it has been very difficult because I live far away and the expenses are high for transportation to come here (Lalgadh Hospital) (Lal P4) |
| Finding | Cognitive dimension (C) |
| Illustration | Health beliefs: Leprosy I am not sure. Maybe it is a curse; it is due to any bad deeds in my previous life. (LalP4)  As I was working in Madras, where the temperature is very high. I also ate a lot of spicy food. I believe my disease is due to this, the heat and spicy food. (LalP10)  Knowledge and health-seeking behaviour: It’s been almost 5 years. I did not know about my disease at that time. Only after it became unbearable I came here. (BPKP11) |
| Finding | Treatment (C) |
| Illustration | If I do not take the treatment, maybe I will be handicapped, have deformities, maybe death. (lalP2)  Self-care practices: I wear gloves while handling hot or sharp objects. I also wear my slippers or shoes while walking. I have to be extra careful while doing my work. (ItaP12) |
| Finding | Psycho-affective dimension (C) |
| Illustration | This is a disease where you do not know what will happen. As it can damage my nerves, eyes, ears, so it is a bit stressful.” (LalP1)  I wish not even my enemies to get this disease.” (LalP2) When I got this disease, my family members were disgusted by me so I left my house. (BirP7) |
| Finding | Patient projects (N) |
| Illustration | Patients presented difficulties addressing this line of questioning (culturally inappropriate?), evoking marriage and the improvement of housing conditions |
| Study: SELF-CARE ACTIONS OF PEOPLE WITH LEPROSY 2014 [8] | |
| Finding | Leprosy complications/sequelae known by leprosy patients (C) |
| Illustration | This disease can cause problems and complications. In case of late treatment, it can bring complications like loss of limb movement. (José)  You may also have, lose sensitivity. You can hurt yourself... You can hurt yourself and feel no pain. (Júlia) It can lead us to lose a foot, a finger, a hand, a leg. (Osvaldo)  When I realised the disease, I was already in the middle, with a small spot. (João)  My eyesight got worse because I am blind girl. I have cataract and she [the physician] said that corticosteroids affect it [...].(Maria) |
| Finding | Self-care actions taken by leprosy patients (C) |
| Illustration | Avoid the sun when it is most intense... use sunscreen... moisturize the skin. (Osvaldo)  The eye drops, because it dries the eyes a lot. (José)  You have to always be doing physical therapy, she said to pick up a ball and keep squeezing it in my hand, because sometimes my hand falls asleep. (Júlia)  Do not walk barefoot. (Carla)  I just comply with the medication. (Francisca)  Feeding must be on time. (Mateus)  [...] do not drink alcoholic beverages. (Marcos) |
| Finding | Possible contributions of a self-care group for leprosy patients (C) |
| Illustration | We want to know more, you know. Because when we know, we will be more careful. We will be more careful. I went through many exams with physicians because I did not know. (Mateus)  I think the government has to adopt a more insightful policy. [...] As I have certain research resources, I rushed into treatment and it was practically a mild form. (Marcos)  Because [she/he] provides more advice, explains more things. We have more information. [...] A conversation in which the person explains that this is not a big deal, as many people tell. We know that prejudice exists, but we have to try breaking down these barriers. (Maria)  The more people have information, the better. It facilitates treatment adherence. You know, the more information, the better. (José)  Because what I went through, I wanted to report it to other people, tell what was good... (Joana)  I am interested because I want to win and I feel good by seeing the patient as my niece and another patient there who had won. (João) |
| Study: Meaning of leprosy for people who have experienced treatment during the sulfonic and multidrug therapy periods 2015 [9] | |
| Finding | Spots on the body: something is out of order (C) |
| Illustration | When those spots started to arise on my skin I asked her (wife) to separate from me in bed, all separated (ES4)  I knew it was leprosy (...). I knew it caused those spots and I could not poke it (ES7)  I saw on television that any patch can be leprosy, and then I went to the doctor (physician). Suddenly a spot appears on your skin so you get very worried(EP1)  I had headache and aching spots. She (wife) told me to go to the doctor and I answered: Ah, make me a tea (...). I didn´t want to go to the doctor because I was afraid of losing my job...(EP3) |
| Finding | Leprosy or hanseniasis? (C) |
| Illustration | I knew of it (leprosy) at the end of treatment. At first, he (physician) did not tell me anything (...) he told me after about three months (...). Well, maybe he had told and I did not understand (ES7)  I was playing soccer (...) and a doctor passed by and saw that I had a spot on my body that would not sweat (...) he applied the warm and hot test (...) after they put me into a police car and drove me to the leprosy hospital (...). Back then it was a dictatorship time, it was not called hospital, it was called leprosarium (ES8)  She (medical eye doctor) got mad, she said: what a nonsense, believe it, this is Hansen´s disease. This is leprosy (...). What a nonsense, you have been untreated, so you are spreading it to population (...) she did not say it was leprosy (...) she just made a fuss (...). I got worried, I got scared, I told it was not my fault (EP5) |
| Finding | Inclusion of leprosy patients as members of the Movement for reintegration of leprosy patients (C) |
| Illustration | About the current treatment, I know it is short. However, I do not know the medications or anything else (ES3)  I always attended (at MORHAN) the lectures and I always talked (...) if I saw the spots I recommended to visit a doctor (ES7)  I jointed the MORHAN to end up this prejudice (ES8) I learned a lot, because what I had read had not convinced me. I talked a lot (at MORHAN). By exchanging ideas, more people came ... (EP2)  I do not know how to get contaminated (hanseniasis). I think I got it at the bus because I did not use to leave the house (...) I'm horrified, because a person (...) gets off (get off the bus seat) and another longer sits down. Sometimes the seat is so warm ... (EP2)  I learned more on what leprosy was (at Morhan). There were people who had the same disease (...). I do not know how to get leprosy (EP3) |
| Study: Experiences of people affected by leprosy in the health services: A hermeneutic approach 2019 [10] | |
| Finding | The disease makes life unsettling and painful (C) |
| Illustration | I did the 1-year treatment, which is multibacillary. After that 1 year, I’m still being treated up until now, because I had various reactions and neuritis. In fact, I’m here today because my foot and hand are swollen. Man, 40 years old. Secondary HU.  […] I already had neuritis. […] I stopped playing soccer […] So, my life stopped. Then, I went to the INSS; they sent me to the INSS, understand? I used to receive a monthly wage, for example, of BRL 1,400. […] It dropped to BRL 900 with INSS. […] it’s turned my life upside down! It’s still rather complicated because I’m not able to work. But […] you have to flow with it. Man, 29 years old. Secondary HU.  Life is normal because I do things, work, look after the kids, drop them off at school, walk normally. Woman, 44 years old. Secondary HU. |
| Finding | Turnover of professionals causes insecurity during the evolution of the disease and treatment (C) |
| Illustration | ‘[…] Because the nurses change a lot. […] I get here and there’s another nurse. The one I had has left and there’s another one. Then, I have to pass on to him everything that has happened this whole time so that he can care for me. […] It’s because the other nurse left and a new one came in. So, I have to tell this one everything that has happened so that they can be aware […].’ Woman, 38 years old. Primary HU.  It wasn’t the same doctor, but another one, and that always complicated things a little. They didn’t have people for that problem […]. The nurse that knew left, but I don’t know where she went! So, the other people were a little lost. Because the right thing would be for the nurse to watch you take the medication. […] Sometimes, I didn’t even take it there, I took it at home, understand?’ Man, 56 years old. Primary HU. |
| Finding | To protect themselves from prejudice, people with leprosy adopt attitudes aimed at reducing social tensions (C) |
| Illustration | […] My daughters also asked: “Is this contagious?!”.  […] My friends ask me a lot of questions about these little red spots, some people even call me a Dalmatian. Man, 70 years old. Secondary HU.  You can’t be completely open with some people that you have this, because they think you’ll transmit the disease to them. Woman, 24 years old. Primary HU.  I’m afraid to even get near her (pregnant woman). What a pain! […] First, there’s the word leprosy. If you say leprosy, people already think you have it. So, not everyone knows I have leprosy, much less where I work, because if they knew they would have had to do a test before I joined the company, and they didn’t. I’m afraid about them finding out […]. If you say the word leprosy, people give you a strange look. Man, 34 years old. Secondary HU. |
| Study: Exploring the Complexities of Leprosy-related Stigma and the Potential of a Socio-economic Intervention in a Public Health Context in Indonesia 2016 [11] | |
| Finding | Socio-economic Consequences of Leprosy (C) |
| Illustration | I couldn’t walk, so scary. I went home right away and I did not want to go work anymore (Female, FGD with affected people).  It is difficult if we do not have money... rice, money, any kind of help is good (Interview with a person affected by leprosy).  Help in funding.... I don’t have money to start a business... If I have enough money, I feel free (Male affected by leprosy, In-depth interview).  There are many things I need. Groceries I need, money I need… to avoid people mocking, we have to run a business” (Female affected by leprosy, Interview) |
| Finding | Barriers in the Health System - and suggested Strategies (N) |
| Illustration | there was sometimes a misdiagnosis. Instead of leprosy, the initial diagnosis was diabetes or arthritis. This means that the start of treatment can be delayed, increasing the risk of disability and prolonging the risk of infection in the community.  people affected by leprosy said that they did not receive information about the cause, ways of transmission and contagiousness of the disease from the leprosy officer.  In a few cases, people affected by leprosy had to pay for the seemingly free leprosy services. |
| Finding | Barriers related to Knowledge, Beliefs and Attitudes in Society - and suggested Strategies (N) |
| Illustration | Many of the study participants lacked a clear understanding of leprosy. |
| Finding | Barriers related to Emotional and Physical Consequences of Leprosy - and suggested Strategies (N) |
| Illustration | One respondent referred to leprosy as a “secret” disease and explained that this is because “it may cause shame”. |
| Study: Stigmatisation and discrimination: Experiences of people affected by leprosy in Southern Ghana 2017 [12] | |
| Finding | Lack of Knowledge/Myths about Leprosy and Stigma (C) |
| Illustration | Many people, including my family members do not believe I am cured because of the deformities, especially the sores on my leg ::: people stigmatise and discriminate against people with disability ::: because of the disease, my wife divorced me and left with our children ::: I do not have contact with them. (Male with visible impairments).  Some public transport drivers refuse to pick us when they discover our deformity. If we go out to shop, some people snub us ::: many people do not want us to be close to them. My husband and children left me because of this disease ::: there are many negative things associated with this disease, but sickness is sickness, everyone has one sickness or the other. However, if we compare ourselves with those not affected by leprosy, they are better than us. (Female with visible impairments)  My family did not care about me, even getting a place to live and food to eat was a problem ::: I became very sad, bad thoughts came to my mind and I felt like ending my life ::: . |
| Finding | Access to health services (C) |
| Illustration | Anytime we were referred to Korle-Bu, we felt uncomfortable because we would be asked to buy our medication ::: with our condition, we cannot work and therefore cannot afford to buy medicines. We feel more comfortable with the doctors and nurses at the Leprosarium’s health facility, where services are free. If we fall sick and the health professionals here refer us to other hospitals, we would rather find money to buy non-prescribed medicines from pharmacy shops. (Female with visible impairments).  When we go to the big hospitals, it is very difficult for us because many doctors and nurses there are scared of the sickness, they don’t believe we are fully cured because of our deformities ::: |
| Finding | Access to employment (C) |
| Illustration | The money we receive from government is not sufficient. This is a big problem because I do not have anybody to support me financially ::: If I wake up in the morning and I have no money on me, I borrow one Cedi to pay for my lorry fare to Lapaz or Nyamekye (communities near the Leprosarium) to beg for alms for two or three hours and I get some money ::: (Male, with visible impairments).  A man saw me on the street begging for money and he asked if I could work ::: I replied that if he had a job for me, I had the strength to do it. He promised to offer me a job but I did not see him again. Potential employers are often reluctant to offer us jobs because of the disease. Even though we are cured, they think the disease is contagious ::: they employ other people even if we can do the job better. (Male, without visible impairments). |
| Finding | Contentment with the leprosarium (C) |
| Illustration | People stigmatise and discriminate against us all the time so I do not plan to leave this place ::: before I moved in here, people in my community always said bad things about me because of my visible impairments. I felt compelled to move to this Leprosarium in order to live with people with a similar condition :::  I have no family outside this Leprosarium. Because of our visible impairments, many people do not want us around them, especially living with them in the same house ::: even if we had money to pay rent, landlords were not willing to accept us since our own family members were unwilling to accept us. We will continue to reside in this Leprosarium because we are happy here. |
| Study: 'Money is the vehicle of interaction': Insight into social integration of people affected by leprosy in Northern Nigeria 2010 [13] | |
| Finding | Prejudicial attitudes (N) |
| Illustration | The commonest negative attitudes identified by people affected by leprosy during the study were avoidance, devaluation, and segregation by family. |
| Finding | Financial independence and income-generation (C) |
| Illustration | The loan has increased my independence. In the past, I used to wait for my husband before I could buy something, but now because of the loan that I use for trading, I can do some things on my own, I don’t have to wait for my husband to do all the things again and it has also brought wealth for enjoyment. |
| Finding | Accessibility to local services (C) |
| Illustration | It has improved my access to places. Where I couldn’t go before, I can now go; you know ‘money is the vehicle of interaction’. I am now going to places like church and contribute my own share. Yes I can now enter many places in the community, but in the time past I was not allowed into some places because of lack of money to contribute to community activities. |
| Finding | Desire for acceptance (C) |
| Illustration | Although we enjoyed the previous loans, we think more loans will increase our recognition with people, so that when they talk, we can talk too:::we can be like other people. You know a little more money like N20 000–30 000 [US$150–200] will uplift us. A lot of changes are noticeable. We and the ‘healthy’ can now sit in the same place to eat together. Our interactions are like those of ‘healthy-to-healthy’:::when we travel to neighbouring towns, we are not shunned any longer. |
| Finding | Dignity (C) |
| Illustration | It has increased my dignity because I am now able to send my children to school and I am able to work like other women in society (P1 Female). |
| Finding | Components of SER that reduces stigma (C) |
| Illustration | Micro-credit loans was the foremost component of SER for stimulating positive community attitudes and social interaction. |
| Study: Changing stigmatisation of leprosy: an exploratory, qualitative life course study in Western Nigeria 2019 [14] | |
| Finding | Connotations and significance of leprosy (N) |
| Illustration | The intense shame and disgrace associated with leprosy. Leprosy was associated with immoral behaviour. Leprosy was also associated with filth. |
| Finding | Causation and transmission of leprosy (N) |
| Illustration | Leprosy was caused by supernatural affliction. Leprosy was hereditary. Natural causes of leprosy included air-droplet infection in poorly ventilated houses, casual contact with skin lesions and ulcers and sharing cups, plates and beddings |
| Finding | Document analysis - 3 sources of stigma (C) |
| Illustration | 1. health promotion messages in primary school books  2. religious teachings about leprosy  3. campaigns by the leprosy service in 1950s |
| Finding | Perspectives on symptoms and signs of leprosy (N) |
| Illustration | Missed early symptoms and signs lead to late detection as failure to associate leprosy without deformities and inflamed reddish skin lesions |
| Finding | Help-seeking by affected persons (N) |
| Illustration | Primary help-seeking pathway for most people was consulting traditional healers followed by referrals to a hospital causing delays in diagnosis and treatment. |
| Finding | Cultural understanding of treatment and cure (C) |
| Illustration | When a person is ill, they are expected to submit themselves for care and during this process of care they are given some medicament. It is the receipt of this medicament that we call treatment. Without receiving the medicament, they cannot progress to a cure. If they do not take the treatment, they will not be cured. Concerning leprosy, we can tell when someone is taking treatment, because we notice changes or differences in their body. For instance, if they had visible skin signs, these signs will begin to clear as they take their treatment. And we know that they are cured when their skin signs have completely disappeared, and they also feel well. So, cure is the complete removal of the signs of the disease from their body and blood. (Male, Farmer affected by leprosy) |
| Finding | The changing stigmatisation of people with leprosy in West Nigeria (C) |
| Illustration | It is possible to regain full acceptance when one has been treated for leprosy. I was shunned before I received treatment for leprosy. No one invited me to participate in family or communal activities because they thought I would infect them with leprosy. But after I was treated and discharged from MDT, they have welcomed me back into the family. I was also recalled by the community. Now we all participate together whenever there are ceremonies such as marriages (Female, Food vendor affected by leprosy). Those who have a means of survival are accepted back in the community. Once they can contribute financially to the community, they’ll be accepted.... If they have money, they will be highly regarded by the community. If they have some change (i.e. money), they will have a voice in the family and community. Those who don’t have money are not reckoned with…even if they don’t have a stigmatizing disease (Female, Trader affected by leprosy). |
| Study: Work and Leprosy: women in their pains, struggles and toils 2018 [[15] | |
| Finding | The pains of leprosy (C) |
| Illustration | My legs ache, right, the nerves, the arms. (Inter. 1)  I was going to work, I was feeling strong pains in my legs, and I even got to get away with leg pains ... (Inter. 3)  If I sit for a long time I feel pain, if I stay a long time I feel pain. (Inter. 9)  It’s difficult for us to talk because it’s not everyone who accepts it, there’s a prejudice right ... I did not want my family to suffer [...] Then only my boss who knows, my manager, that I told for them and they said that they would help me, that I did not need to tell my co-workers, that it could be between us ... I was even scared, because leprosy, for those who know it is also the old leprosy, then everyone was afraid to touch things ... I said “there the girls will not even want to sit next to me” [...] and I will not tell, not to spend all the time wondering. (Inter. 6)  To leave that neither I went out, to jump these things, already in one, in a taste; I was homelier; I insulated myself in the house. (Inter 9) |
| Finding | Changes with the disease and adaptation at work and daily activities (C) |
| Illustration | So, when they knew, they immediately sent me to the work doctor. And the doctor at work immediately removed me from handling food ... Then they put me in the refrigerator sector, which is where the yogurts are, those things there ... That is food that is already packaged, you know? [...] And I began to feel the pains in the legs, for climbing stairs only to get products... (Inter. 3)  [...] I never managed to twist clothes, and I never could do the washing very well ... today I already explain, I will wash it my way, and it has to be a softer brush, so it cannot be that brand new brush. (Inter. 8)  The new job I got, I went in there, I explained to them that I was in treatment, that it was no longer ... transmissible; the doctor made a letter saying it was not, then the company’s doctor accepted and I started to work. (Inter. 9) |
| Finding | Being a woman with leprosy (C) |
| Illustration | I think that the fact that we are women and have a lot of faith in things, having to believe, having to raise a child and such, then you accept things and you end up carrying on ...(Inter. 8)  No. Boys, not men. It was Always me who did everything .... they used to work in the countryside and then helped in the field. At home, it was all with the girls [...] Only the men who did not used to help at home, but we worked on the farm, and also worked at home [laughs]. Now at home, I do not know if it’s because we were young and also women, we did not need the men to be helping. (Inter. 5) So, I do not think so. I think this disease there, when you take a person can be a man, a woman ... if you do not treat, you will be deformed. [...] then I think it goes from the head of each one. This man-and-woman business has no difference. (Inter. 9) |
| Study: The dynamics of stigma in leprosy 2004 [16] | |
| Finding | Strategies of concealment caused by expected stigma. (C) |
| Illustration | I had seen beggars with Kustha Rog when I was in Calcutta. When I got Kustha Rog myself I used to feel scared thinking about those other people who had Kustha Rog. . . In our village there is a saying that if one of the villagers gets this disease and if another person is close with the infected person, or sits with, eats the jutho (a person’s food leftovers) of, or if the husband, or the wife has the disease, then the other person will be infected with the disease. . .  In my village the people do not tell me not to walk with them, sit or eat with them, they did not say or do anything. My disease is not clear and that is why most of them could not find out. The diagnosis of leprosy was seen as a trigger to discrimination. Felt stigma led to a strategy of concealment. I did not tell my family about the disease. I was afraid they would be tense and get worried. We do not have enough food to eat and clothes to wear. I thought that whatever happens, will happen to me only. |
| Finding | Strategies employed in managing experienced stigma. (C) |
| Illustration | The villagers, my neighbors know about my disease. Sometimes when I get into an argument people say bad things to me, like “you have Soon Bairi” (leprosy). At that moment I feel really bad, and keep quiet thinking ‘what to do if I do have that disease.  No one in my family ate with me for at least two to three months, because they thought this disease was contagious. All of them felt bad and advised me to take my medicines continuously . They said that taking the medicines could cure my disease. |
| Study: Leprosy Resilience with Disabilities Due to Illness: A Qualitative Study 2020 [17] | |
| Finding | Self-stigma (C) |
| Illustration | I stopped participating in activities with my neighbors. I prefer to remain at home. I began to reduce activities with people other than my family. I felt uncomfortable even though their attitudes did not change. I am ashamed, feel unworthy when my body is full of disease, especially when someone visits. I feel ashamed, not confident to gather with neighbors after leprosy because my body's skin has turned black. |
| Finding | Psychosocial problems (C) |
| Illustration | I often have difficulty falling asleep at night, sometimes fearing that the disease will get worse. When I was first told that I had leprosy, I always felt afraid I would not recover from this disease. |
| Finding | Active coping (C) |
| Illustration | Thank God, the family supported me during the treatment, taking me to get medicine. The neighbors also did not change their attitude; the officer also always visited me. Sometimes delivering drugs. Every time I finish praying, I always ask that I get well soon. Now I work in the garden, if I have a lot of thoughts I usually go to the fields. The air is fresh, lots of greenery. I often calm down with activities in the garden. |
| Finding | Positive adaptations (C) |
| Illustration | Now, I work in the paddy fields of my father. The results are quite good. If the harvest is good, it can benefit a lot. For three years I worked, the results were good every harvest, which was planted in all kinds. Rice, corn, tobacco, and red onion. My family and I often gather at night. Especially if there are guests, usually until midnight, telling each other's activities. After recovering and completing treatment, I feel better. I'm comfortable when I'm active. |
| Finding | Characteristics of resilient individuals (C) |
| Illustration | Yes, even though I am handicapped, many neighbors still use my services to plant rice seeds in the fields. I am also always willing. I can deal with the problem calmly. Not rash, the point is already received. If there is something that is not what I want, it is not too angry or not shouting as before. From the break of dawn, I went to the rice fields, mowing the grass until 9pm. resting at home. In the afternoon, I was in the fields again mowing the grass. I am capable of grazing cows with two heads of RT. After a year, there will be profit sharing. If for every day I eat from the results of the farm. |
| Study: An assessment of women’s empowerment in mixed self-help groups in Dhanush district of Nepal 2020 [18] | |
| Finding | SHG women are empowered (C) |
| Illustration | Before, I used to think whether to be alive or to die. But I am cured. Now I think to run a business or to raise goats, cows.... – 62 year old SHG participant  Now, my husband earns money and gives it to me to spend as needed for the family. I do everything myself. When my husband is not there, I have to be the guardian.... – 35 year old SHG participant |
| Finding | SHG women know their rights (C) |
| Illustration | Rights are equal now. Before, women used to be kept in under pressure in the house. There was no trust towards them. Always a question was put around them such as how can someone’s wife or daughter go out? How could a daughter or daughter-in-law be engaged in self-employment? How could they talk with other men? People think badly of them but now they are more open. Now they can talk and have less criticism directed towards them. Even their guardians could not criticize them because they understand that going out can make them knowledgeable, and help them learn about the environment, as I did when I went out.” |
| Finding | SHG women engaged in income-generating activities and saving schemes connected to SHGs (C) |
| Illustration | After taking 24 months of medication, I became cured. I have started income generation activities effectively. Initially, we were given Nrs. 20,000/-. Now we have about 500,000/-profit. People are happy with me to see my progress and that I repaid loans as well..... |
| Finding | SHG women reported increased levels of self-confidence and self-esteem (C) |
| Illustration | Before, I was afraid to ask the teacher about the progress of my children’s education. Now, I can ask confidently.... – 28 year old SHG participant |
| Finding | SHG women participate in social programmes - opportunities in the planning and decision-making tasks (C) |
| Illustration | when a woman is mistreated by her husband because of leprosy, I go to help that woman to show somebody is there to care for her, and I counsel her husband as well... |
| Finding | Barriers in participating in mixed SHGs (C) |
| Illustration | Women have many things to do like cooking, household chores, and childcare. And men come and order food at home. Women continue with various household chores, cooking meals, looking after the cattle and cutting grass. They just work all the time. The main barrier is that people stop us from participating in the groups led by leprosy- affected people. They said that leprosy will transmit to us as well... – 32-year old disabled participant |
| Finding | Barriers to women's empowerment in mixed SHGs (C) |
| Illustration | Male dominance: When we attend other meetings then men comment on us and say that now men have no value, only women have... – 35 year old SHG participant  If a woman participates in group meetings, people in the community suggest that she is having illicit relationships with other men.... – 55 year old SHG participant.  Lack of assets and resources: Men do not allow women to go out. We have to talk and fight for women’s rights. If we won’t talk, we will not get rights...” – 67 year old SHG participant.  Social and cultural barriers: “If anything happens in the village, women are not allowed to go – only men can go...” – 35 year old SHG participant  “There were restrictions on newly married women; they were not allowed to show their faces. It used to be a big problem at that time. Their parents-in-law used to beat them...” – 32 year old SHG participant. Lower capacity: We cannot be a leader because we are illiterate, so we cannot understand the things that are said or written – 50 year old SHG participant |
| Study: Disease experiences of female patients with Hansen's disease residing in settlement in Korea 2020 [19] | |
| Finding | Inescapable shackles (C) |
| Illustration | My knee became flaky one day and my elbows the next day. It did not hurt at all, but it kept leaving a scar. Back then, I had eyebrows, hands, and feet... People didn’t recognize me because my face became  swollen and my appearance changed. (#2)  After the face became swollen, deep wrinkle-like lines formed and face became bluish and fat from being swollen... My eyes were lifted and only my eyes became frighteningly large, making me ugly. (#4)  When food was brought to the room, that’s all I ate and I could not go outside. If I really wanted to go out, I had to go out at night. Everyone was in a tough position because of me. My younger siblings are just as big a victim as I am. Our neighborhood used to share a common well, but they wouldn’t let us drink from the well. (#5)  I want to work outside, but it is too much of a hassle to go outside, so I stay here doing the laundry and feeding the pigs. (#7) |
| Finding | Suffered as if being in prison (C) |
| Illustration | I was living hidden in a small room, but the local police came and said that there is a good place where they could give me medicine and treat my disease, so I left the house with them to be hospitalized. (#9) When I left the house to go to the hospital, I had many thoughts about whether I am going there to live or to die. I was leaving to treat my disease, but it was quite scary to be away from my parents. (#10) Being in captivity, there is definitely a sense of captivity tied to being on an island. Not being able to go out freely, it is suffocating. There were some people who died by drowning while trying to swim to land for freedom. There were others who got imprisoned for getting caught while trying to escape by boat at nighttime. (#3) |
| Finding | In no position to be a woman or a mother (C) |
| Illustration | Some lady told me to live with that guy because I’m lonely. I got married when I was 19 years old. I didn’t know what I liked or what was good, but because I was so lonely and hungry, I lived with him with a sense of having someone to rely on. (#6)  Men kept asking me to marry them. But when I told them I didn’t want to, they made me kneel and forcibly... It makes me think that was not a person should live. (#10)  I became pregnant and gave birth at the hospital. After 9 months, they sent my child to foster care. Afterwards, they forced me to have the surgery. They call our children “uninfected child.” They say our children haven’t been infected yet, but will eventually be infected, so we should send them away. A baby who hasn’t even been weaned off breastfeeding. (#4)  I always that I’m a woman, so I should be pretty. It bothers me that whenever I go out, people run away. (#6) |
| Finding | Another hometown (C) |
| Illustration | People didn’t do anything at first, but they would move away after seeing my face. Even if some people got angry at me, I couldn’t say a word even though it was so unfair. I’ve lived to this point with my head down. (#7)  I went out into the main society, but I couldn’t live there. People don’t say they like it or don’t like it, but I think to myself about me being a leprosy patient. (#8)  Here, we can talk about each other’s situation. In the outside world, we can’t talk about ourselves. That’s why it’s difficult to get close to others, so this place is most comfortable. (#1)  Here, everybody is family. We have stepsons, stepdaughters, stepparents, brothers, and sisters. We sympathize with each other. Even though my hands are like this, there is nothing to be embarrassed about. (#10)  My mind is peaceful and happy. I don’t resent anything now. If things stay the way they are and my time comes, I would be satisfied. I have nothing more to wish for. (#3). |
| Study: Improving treatment outcomes for leprosy in Pernambuco, Brazil: a qualitative study exploring the experiences and perceptions of retreatment patients and their carers 2021 [20] | |
| Finding | Personal factors: Knowledge and Information quality, health beliefs, psychological impact and character (C) |
| Illustration | I never shower with hot water and then suddenly with cold, or after eating, because I thought that’s what causes leprosy. I don’t know how I got it. (018 – Patient)  ... I know the woman in the waiting room. She told me about her nerves. They became defective. Others I know are on crutches, they can’t walk. (001 – Patient)  ‘I could have stopped taking the pills. But the clinic staff kept saying, "Don't stop, otherwise it comes back even worse.". So I kept going.’ (010 – Patient)  ‘My beautiful legs, my lovely feet, suddenly looked bruised ... If there is no cure, I will jump off a bridge. Because I will not live life sick with this leprosy, like a loser. I just want to be the same as I was.’ (018 – Patient)  ‘My husband was scared. He was afraid of having sex ... my daughter said, “Mom, if you have leprosy, you have to separate.”. She separated her glass, her plate, everything.’ (026 – Patient) |
| Finding | External factors: Socioeconomic factors, Structural factors, Support factors (C) |
| Illustration | I worked in grape farms. Today, I don’t have the courage to work anymore, under the hot sun ... I am too weak. But if I don’t go to the farm, how will I feed my children? Their lives will suffer. (001 – Patient)  Who will hire a person with leprosy? There is prejudice. I need to heal. I need to be cured. Because I have dreams, projects ... (026 – Patient)  I told the doctor I felt very weak. She did all the tests quickly, found out I was anaemic and gave me medicines for it. (018 – Patient)  It is difficult in the countryside. There is a lack of awareness. Health care professionals need to visit us at home because it is hard travelling to clinics. (005 – Patient)  Only my eldest boy works. I have another boy at school. He needs me as a mother, but I am not well. (019 – Patient)  My neighbour said, "Get away from him, that disease is transmissible!". So I isolated myself, but then, when his wife and children came out to talk to me, I told them, "No, I am not infectious.". (002 – Patient) |
| Finding | Clinical factors: Treatment and side effects, experiences of diagnosis (C) |
| Illustration | It’s hot here every day, and I’m exposed to the sun because I’m a farmer, so my skin became dark because of the medication. (019 – Patient)  Since the disease has a cure, you have to try to do everything to make things better. (016 – Patient)  From the second day onwards, it was only improvement. (012 – Patient)  I have already been treated for two years and I still have the leprosy. Where is there result? I wanted to stop taking the medicines but the clinic staff said that wouldn’t be good. (010 – Patient)  The hospital told me it was rheumatism. It got worse so I went to the clinic again. Then they told me I had advanced leprosy. If they told me sooner, maybe I would have suffered less. (004 – Patient) |
| Finding | Healthcare professional-patient-carer relationship (C) |
| Illustration | ‘I learned a lot from the doctor. She said there are five types of leprosy, and mine attacks the nerves and causes me to have reactions.’ (002 – Patient)  I was surprised. How come the doctor stopped the medication if I still had a lesion? It bothers me a lot that I stopped the treatment then. Nobody told me exactly why. (019 – Patient)  In the eyes of the doctors, I am better, but my leg still feels numb, that’s the problem. (002 – Patient) ‘The two doctors here are very good. If the doctors are worried about your health, they will find out what is wrong as soon as they can.’ (026 – Patient) |
| Study: Practices for self-care in Hansen's disease: face, hands and feet 2018 [21] | |
| Finding | Knowledge and execution of self-care practices in Hansen’s disease (C) |
| Illustration | I can’t stay in the sun, I spent some time in the sun and got black. (Interviewee 5)  [...] In the skin, I was [advised] to use sunscreen when I go out, not to stay on the sun [...] (Interviewee 6) I arrived with dry skin... then she [nurse] said: you have to moisturize! But I didn’t think it was because of the treatment. (Interviewee 24)  For the eyes she gave some guidance, that I went to the ophthalmologist, be careful with them, right, I always were sunglasses. (Interviewee 14)  She told me to keep the nose clean and be a little more discrete when sneezing (Interviewee 21)  To avoid cuts, injury to the feet, because it feels numb, with the sequelae I got I can’t feel my feet, for instance I hit it 3 months ago and didn’t feel it. (Interviewee 16) |
| Finding | Singularities and challenges in the self-care for leprosy (C) |
| Illustration | During the day I can’t because I start work very early, I have to care for the children and I don’t have time to care for myself, and that’s the type of time I want to have, to care for my health, for my problem, so it doesn’t get worse. (Interviewee 16)  I have a hard time wearing the glove because I think I put it in every finger and then when I look two fingers are in the same finger-hole [...] It’s difficult for me to hold things, coins, I can’t write, my writing also got too ugly. (Interviewee 5)  Doing these practices is no trouble at all... if its for my good there’s no problem. I don’t wear sunglasses cause I’m not used to them, I don’t like it. (Interviewee 23)  About the disease? I don’t even know because she didn’t explain. She said it was a skin condition and that was it, she ordered medication, and every month I come and get it. (Interviewee 20)  That it causes skin patches if you don’t care for it and that can be harmful right... so the treatment is caring for your- self. (Interviewee 9) |
| Study: Development of a rights-based counselling practice and module to reduce leprosy-related stigma and empower people affected by leprosy 2017 [22] | |
| Finding | Health perceptions (N) |
| Illustration | There were different understandings related to the cause of the disease (‘genetic’, ‘black magic’, ‘touching something dirty’). Many people affected by leprosy did not realise they were no longer infectious after starting multi-drug therapy (MDT) treatment. |
| Finding | Feelings and emotions (C) |
| Illustration | I was very embarrassed when I found out I had leprosy so I never tell others. Keeping silent is better. (IDI 8, woman, 50 years)  Sitting in the room, I know it is nothing [I know it is not good], I should go for work, maybe later if I am cured. (IDI, male, 23 years)  I am very sad and afraid, I feel alone, I feel a distance from my family, actually I like to help my family cooking but I am doubtful. (IDI, female, 28 years) |
| Finding | Human rights and discrimination (C) |
| Illustration | I used to run a small business, selling toys. Because of leprosy, I lost my customers and nobody buys my toys. Hence, my business is bankrupt. (IDI, male, 35 years)  I stopped going to school because of my leprosy. My friends are always making fun of me. I feel ashamed and uncomfortable going there. (IDI, child, 13 years)  Because of leprosy, my wife keeps away from me. She does not allow me to hold my granddaughter ::: I am sad but I do nothing. (IDI 53, male, 60 years) |
| Finding | Role of family, friends and neighbours (N) |
| Illustration | the family of the person affected by leprosy is affected by the stigma associated with it - a positive influence on the situation by giving care and support or reinforce stigmatisation, for instance by keeping their distance or by encouraging the concealment of the disease. |
| Finding | Key issues identified during pilot: individual counselling - positive response to discussion on rights, family counselling - changing patterns of stigmatisation in families and group counselling - realise they are not alone and there are others with same disease (C) |
| Illustration | Yes, you are right. I cannot just sit and wait for help. I should do something with my future like earning money. (PCN53)  I have talked to my teacher about giving me more time [to complete assignments] since I have to write with my left hand. (PCN20)  We take care of our child together. My husband now believes that I can do it and I share household tasks with him. He does not treat me as a patient any more, even though he knows I am still taking medicine. (FCN1)  My husband is very busy, he does not have the time. (PCN61)  You just meet me, it is enough! My family doesn’t need to know about this visit. (PCN16)  Let me be very open with you. Talking with you [another person affected by leprosy] motivated me. My perspective was wrong: affected persons still have the right to work. I learnt from you that I should not wait to be declared cured. (PCN, 14) |
| Study: Dealing with stigma: experiences of persons affected by disabilities and leprosy 2015 [23] | |
| Finding | Impact of stigma on emotions: persistent self-image of a sick person (C) |
| Illustration | Why should I share my feelings with my family if I feel they do not care for my feelings? If I share them with people they will avoid (dijauhi) me even more. (Person affected by leprosy 1 IDI)  it cannot be that we are cured; we always feel pain in our muscles. We have skin patches and they never go away. (Person affected by leprosy 7 IDI)  See my left foot, it is crooked, it is abnormal, and it is sick. (Person affected by leprosy FGD1)  I always wonder when my left foot is going to be cured and become a normal foot again. (Person affected by leprosy 7 IDI) |
| Finding | Impact on thoughts: limiting social participation and employability (C) |
| Illustration | when I attended an interview for recruitment the interviewer noticed the skin patch on my face. I told them I am cured from leprosy, but they did not trust me. I even showed them a formal letter from the community health services but they rejected me. (Person affected by leprosy IDI 1)  Another participant added: I have to earn money for my children. Before I got leprosy, I worked as a cleaner, washing clothes, sweeping floors, and cooking. A few months ago, I was finally cured from leprosy, but nobody wants me to work in their house; people avoid me and ridicule (mengejik) me because of my crooked hand. (Persons affected by leprosy FGD 2)  Business communities have a prejudice regarding my impairment. When I applied for a job, they spontaneously label me saying I look as somebody who is looking for charity. (Persons affected by leprosy IDI 1)  We know we are sick because people have told us so. As sick persons, we have no friends, no hope, and no future. (Person affected by leprosy FGD1) |
| Finding | Impact on behaviour (C) |
| Illustration | I do not know what I should do. I prefer to sit or walk around the house. If my family asks me for help, I help; if not, I usually just sit. (Person affected by leprosy FGD1)  Keeping silent is better than doing something. Being labelled cannot be stopped and I cannot stop people labelling me. Meeting people, for me, means being insulted (dihina). (Person affected by leprosy IDI 6) We are ill. Going to the community health services, getting and taking our medicine regularly, meeting the health officers if we are in pain, and asking for medical treatment. That is enough. Just to do these actions is enough. (Person affected by leprosy FGD 2)  When we [people affected by leprosy] are bored, we go to the kitchen and wash some dishes, but our family members shout at us and we get insulted (dihina). So we do not want to do it again, because doing activities is more isolating (dikucilkan). (Person affected by leprosy FGD 2) |
| Finding | Impact on relationships (C) |
| Illustration | once, I felt really disappointed when I met my neighbour. When I greeted him and offered him my hand, he rejected my hand without replying my greeting. Then he went inside his house and closed the door on me. He is a strange neighbour and what makes it even sadder is that he works in a community health service. (Person affected by leprosy IDI 2)  If there are no urgent matters, we prefer staying at home. From our experience it is better to not make contact with people. People surrounding us expressly avoid us and they continually label us as patients with a contagious disease. (Person affected by leprosy FGD2)  For me, there is not any benefit in interacting with people. Many times I have tried to start social relations by smiling and saying “hi” to people, but they still look at me as an enemy. (Person affected by leprosy IDI 1) |
| Finding | A perspective on coping strategies (C) |
| Illustration | I have the rights to live so I need food and drink for my life. I need money to buy that, so I need to work. (Person affected by leprosy FGD 2) |
| Study: Adaptive strategy of women's leprosy in indonesia psychic experience of women with leprosy in living a community life 2020 [24] | |
| Finding | Mental stress is a part of of everyday life (C) |
| Illustration | ... I had to live with this long tenure, even though I had to go through many obstacles, especially ridicule from people, so I recovered quickly (P. 10)  . .. I always have to do self-care every day so that it doesn't spread to other people, and I have to provide free time and sometimes miss what opportunities I should get (P.6).  "... everywhere I have to carry my disability ... I have to think again to shake hands with people because of my disability (contracted fingers) (P.3).  I am still young, I don't have a husband, I will give up if no one wants to marry me. I asked myself, is there anyone who wants to marry someone like me? (P. 8).  I am afraid that my husband will find out about my illness, surely he will be disappointed and will leave me. If I take medicine, I have to make an appointment with the health worker, I must recover from this disease without my husband knowing. (P. 11). |
| Finding | Physical conditions that are far from aesthetic value (C) |
| Illustration | I also feel uncomfortable with my skin, which looks dry. Using any moisturizer still looks shiny. But if left untreated, then this skin looks dry scaly (skin on the feet). (P.16).  The sores on the soles of my feet are always watery and smelly, and they smell bad. I am sad when I meet many people. (P,14).  I can only regret, since there are red spots on my cheeks. I am not beautiful anymore. I feel embarrassed, if I meet old friends. (P.13).  I am ashamed to wear jewelry with this deformed hand. It felt like there was something else about me. (P. 12). |
| Finding | Guarantee of happiness that is hard to come by (C) |
| Illustration | Friends who used to be close to me now rarely see me. Maybe afraid of catching it, knowing my illness. (P.8).  My children were unusual in seeing me before. I am sad now, because they stay away from me, since knowing the condition of this disease. (P. 4).  Sometimes I feel that I am not human, so I think that I will leave my home and be away from my family. (P.6).  My family is embarrassed because I have this disease (leprosy). Now my family rarely takes part in an activity called "Jamaah Yasinan", because I am ashamed of the leprosy that I have experienced. (P. 7).  I really wish I could caress, sit side by side and joke with the children, but he keeps avoiding, feeling scared because I have this disease (leprosy), even though I gave birth to him. (P.15).  I was forced not to give my milk (ASI) to my child who is 5 months old. I replace it with formula milk. My baby is being cared for by my husband and oldest child. (P.14). |
| Finding | Looking for a certain point to surrender to God for all suffering (C) |
| Illustration | God gave the disease and I believe God also gave the medicine. For me, this is a test that I must go through, and I still have to try so that I can get the medicine that can cure my disease. (P. 13).  If I die from leprosy, then I will accept it as God's destiny and I sincerely accept it and I must live it. (P.14). For me, this illness that I suffer is a warning from God to me, so I have to get closer to God. (P.11).  The religion I believe has taught me to accept what is inside of me. My religion also always recommends making every effort and praying that my disease will be quickly removed from my body so that it will heal. (P.7). |
| Study: Stigma, deforming metaphors and patients' moral experience of multibacillary leprosy in Sobral, Ceará State, Brazil 2009 [25] | |
| Finding | Repulsive Rat's disease (C) |
| Illustration | I wasn’t afraid to tell anyone I was a Hansen’s disease carrier (...) I didn’t know it carried, well, such a heavyweight of prejudice (...) since I didn’t pay much attention to what the doctor said! It was at that moment I realized that, in reality, Hansen’s disease is called (...) prejudice! |
| Finding | Racist skin rash (C) |
| Illustration | Doc, if it’s going to mess up my body, don’t prescribe these pills, no! I’ve heard that there’s two kinds of pills – just the little white one alone (...) and the little white pill together with the little black one (...) don’t give me that one, no way! I don’t want to become a ‘nigger’ [negão], no way! Before, I was only a leper. But now, I’m going to be a nigger leper [leproso negão]!”. |
| Finding | Biblical curse (C) |
| Illustration | I was diagnosed during a screening at my work (...) I almost died (...) I never thought I’d have this disease! (...) The doctor said it was Hansen’s disease (...) but I heard his nurse say ‘leprosy!’” (grifo nosso). Isn’t leprosy that ancient Biblical curse (...) where your fingers fall off? What I have is Hansen’s disease! |
| Finding | Lethal leukaemia (C) |
| Illustration | I’m as good as can be (...) only these white skin patches [pano branco]. the doctor said I had this disease (...) hansemia He refused to take his prescribed medication _ “They made me sicker and laid me up (...) unable to work”.  See this thing on my forehead and this here on my nose? I’ve had these since infancy! This scar on my left hand is a burn (...) I burned my hand and it stayed like this! |
| Finding | Skin spot day (N) |
| Illustration | Clinic staff publicly scrutinise identifiable patients' skin spots |
| Finding | Stigma: social leprosy of modern times (C) |
| Illustration | We're all lepers! |
| Study: "the Body I Was and the Body I Am": Conceptions of Women with Alterations Caused by Leprosy 2012. [26] | |
| Finding | Aesthetic dimension of the body The beautiful (healthy) and the ugly (sick): significant elements in the construction of representations (C) |
| Illustration | before or now? I didn’t have these “stains” before, now there are always these things appearing, like: wounds. I used to sunbathe a lot, go to the beach...not today [becomes silent] (I19A).  [...] how I see my body now? [talks weeping] Totally unrecognizable. I cry when I see my body like this, because I didn’t have these ugly things before, and now I do. That hurts a lot [lowers her head and voice] it hurts the body and the heart even more (I22M).  Perfect body? Humm... I see that beautiful body, smooth, clean, everything right... without leprosy... like that body I had before this disease (I4M).  I do not find myself a complete woman because of the disease. According to me, a real woman is a healthy woman, in good health. I differ from the others. I am a woman, but I do not longer feel like a whole woman, a woman like I was before (I4M). |
| Finding | Functional dimension of the body (C) |
| Illustration | Being a woman and having one’s body altered by leprosy? I think it’s bad [...], sad [...]. The pain I feel is constant, it’s terrible. Then, if you work, you have to stop working because the problem distances you from work. You need the rest, the diet, all that [...] (I35M).  Woman’s body? Being entitled to my health, being healthy again to be able to work. I am unable to work [pause] because I feel a lot of pain, those things [...] (I31A).  It changed, like, with the disease, which affected my hand and my foot [shows her hands, gets up from the chair and also shows her feet]. I don’t walk anymore like I used to [hesitates]. My hand also changed, lost force, and things fall from it [shakes the hands]. Today my body got really ugly, sick, mutilated [long pause, crying] (I4M). |
| Finding | Living with one's new body and self-care (C) |
| Illustration | although my change is not that intense yet. It won’t get there, with God’s help, it won’t get that intense. I stay alert so that it won’t get there. I can still say: Oh dear, she’s worse than I? .... So, so as not to reach that stage, I have to take the medication and give up some things (I28A).  We have to overcome it, we need to have willpower to overcome it [...]. We have to think that there are people with worse problems than ours, much worse [...]. Sometimes we think that our problem is bigger than other people’s, but we hear people say: ’Oh dear, that...’, ‘Damn’, I’m fine compared to that person, right? (I30A).  I use creams, just for the feet, just for the hands, for the body; I use bath oil. I take care with skin cracks, with marks, because [hesitates] if you like it, you take care of it. So, I [hesitates] I need to take that care with my skin. So, to me, when I see a little stain, I feel agonized; I quickly put on some cream. (I17M). |
| Study: Hansen's Disease Patients' Perceptions on Their Altered Fundamental Human Needs: Indications for Self-Care 2020 [27] | |
| Finding | Physiological needs (C) |
| Illustration | I remember the spots that appeared on my body. Not long ago my back started to get full of light spots and I was very worried. Now it’s getting better. (P2)  The information was very good, I just said nothing about food, if I could eat any kind of food, and I forgot to ask, so I deprived myself of eating a lot at the beginning of the treatment and lost a lot of weight. (P4) The guidelines given by the professionals (doctor, residents, nursing, and physiotherapist) were very good, they explained the disease and treatment. They informed me of the precautions I should take, such as using sunscreen, moisturizer, and healthy eating. Things I didn’t do before. (P3)  About the guidelines, I was informed to choose suitable shoes, not to drink alcohol, not to sunbathe and to keep the skin always hydrated. (P5) |
| Finding | Security needs (C) |
| Illustration | It started with light spots on the legs and back, I had tests, I took to the doctor, but did not certify anything. I went often after consulting with a dermatologist and never got it, meanwhile, the spots started increasing. So, I was forced to pay for a private doctor who requested a smear-positive for Hansen’s disease. (P10)  I use sunscreen and moisturizer, I’m careful with my feet, I wear comfortable shoes because, because of the lack of sensitivity, I already got hurt and didn’t feel it, I just realized it because I was bleeding. I’m careful when I cook too, because of the temperature. I do not want to be discriminated against by anyone. (P10)  So, I started wearing longer clothes and long pants to cover this spot on my thigh. (P9)  I get annoyed because of this I use a sunscreen based to slightly smooth the imperfections on my face and, as I work with the public, it ends up giving a bad impression to other people. (P6) |
| Finding | Love and/or social needs (C) |
| Illustration | There is prejudice and lack of information because Hansen’s disease is an apparent disease and people are afraid to approach. It is considered an ugly disease because it causes deformities in the body, skin, atrophy of muscles and nerves and is biblical. Very much mentioned as a disease without a cure. (P9)  It is a contagious disease. That’s what the hospital staff told me, and I couldn’t be around anyone. I was with my daughter hospitalized and they isolated me from her, and I was already doing the treatment. (P7)  I didn’t tell almost anyone, only close friends and family, because of the prejudice that still exists. People often move away because they do not know about the disease. (P2)  I was afraid of being prejudiced, I didn’t tell, and I walked away from everyone. (P4) |
| Finding | Self-esteem needs (C) |
| Illustration | The imperfection that bothered me the most was that of my face. As I had not before, it drew a lot of attention. I used a lot of makeup to hide it, now it’s almost gone. I was devastated, feeling very ugly. (P4) The Hansen’s disease reactions made me look like a monster. Looking at myself in the mirror horrifies me. (P6) |
| Finding | Self-actualising needs (C) |
| Illustration | I was totally lost at first, I was asking God not to let me lose any limbs. (P1)  Only God to support me and help me in this treatment. Without him, I would have given up on the first reaction. (P5) |
| Study: Perceptions of people with leprosy about disease and treatment 2016 [28] | |
| Finding | Faced with the disease: the first symptoms to confirm the diagnosis (C) |
| Illustration | I was cooking, I craze to mix in the pan, burn me and not see. I went to the health center (E6, F, 61 years). I went to the doctor because it started to give me some spots on the skin, and I thought it could be the reaction to the surgery I had done (E8, M, 45 years).  Back in the 70s, I started to feel a fire in the feet, warmth in the feet and I started doing tests, a doctor who was speaking uric acid, another said it was the column (E1, M, 75 years).  “You will be the last.” Then I thought, my God, I am being discriminated, but no problem (E5, F, 58 years). I did the exam, but frankly I thought I had not, I was sure that I had not. However, then the doctor said, “You have it.” (E6, F, 61 years).  I never expected that this would happen to me.... because any of the family had these things (E7, F, 44 years). |
| Finding | Motivations, benefits and difficulties related to the treatment of leprosy (C) |
| Illustration | Almost a year ago I received the news, I was worried, but then I started having treatment, and I felt better (E2, M, 63 years).  I am doing the treatment right to see how it goes. The back was very bad, very itched that turned up wounds. Now some time now no longer itched. The arms have vastly improved; the spots are fading (E3, M, 51 years).  It is getting better, I take just right, had much numbness in the fingers, and it is improving. I had much pain in the arms; it was all red, and now there is no more, it is gone the redness and the plates (E8, M, 45 years).  I think I am good because now I can go alone, because before my daughter had to bring me (E4, M, 77 years).  One of them has had, and he is now healthy. Moreover, that gives me, even more, strength because I see his skin and is healthy, beautiful (E5, F, 58 years).  I did not stop going anywhere, I am evangelical, go to church, I am well received (E6, F, 66 years). |
| Finding | Family as support or exclusion (C) |
| Illustration | A son who lives in the Home Pr and have a car, he who took me there to the Dr. My family helps in the treatment (E1, M, 75 years).  At first, I lived alone; then my daughter came to live with me. She was watching me at first because I could not walk with pain (E4, M, 77 years).  A brother treated me differently and felt very offended. He said it was to separate plate, cup, forks, spoons because he took. Even when we went to his house, my son and my sister took the vaccine did not catch. I said I was not contagious so because if it were to take had already caught the son and my sister to have more contact with me and they did not get (E7, F, 44 years). |
| Study: The meaning of leprosy and everyday experiences: An exploration in Cirebon, Indonesia 2013 [29] | |
| Finding | Giving Meaning to Leprosy (C) |
| Illustration | I heard that all of their fingers will come off. Is that true? Interview 7: female, age 36)  As far as he knows about leprosy: the face become pale, become introvert and did not want to join in their activity. (Interview 13: male age 62)  I am not shy and I do not have a low self-confidence, so I do not have leprosy right? (Informal interview 1: male) |
| Finding | Seeking Care: Perspectives on Diagnosis and Treatment. (C) |
| Illustration | The moment the leprosy worker did not want to shake my hand, I had the feeling leprosy cannot be cured and that people will not be friendly with me anymore. (Informal Interview 2: male)  An older woman (Interview 18: age 74), however, felt disappointment with the socialising procedure of the leprosy worker as it made neighbours actually more afraid and as a result they avoided her. In contrast, some persons affected by leprosy said they benefited from these visits (Interview 1: male age 20, Interview45: male age 21, Interview 3: female age 45).  They said they were visited routinely at home by leprosy workers who gave counselling to increase confidence and to provide information to their family, who supported them in daily and community activities. Some friends advised me to go see a dukun. . . , she is not solving or healing my illness but asking for more money. Since then I never went to dukun again. (Interview 18: female 74) |
| Finding | Understanding Healing and Cure (C) |
| Illustration | Every disease has its remedy and it depends on God’s mercy. (Interview 28: female 20)  Well, if God listens, then my prayers will be granted and it means I am healed. (Interview 29: male age 36) |
| Finding | Impact of Living with leprosy (C) |
| Illustration | I will isolate myself. . . . Yes, because I do not want to infect my children. (Interview 7: female e age 36) One participant (Interview 35 male, age 41)  who has impairment due to leprosy said that most people more saw him as a body rather than as a human being. . . Hmm like this: do not feel sad or do not feel discouraged. . . you are not alone. (FGD 18 People affected). |
| Study: People like me don't make things like that': Participatory video as a method for reducing leprosy-related stigma 2016 [30] | |
| Finding | leprosy-related perceptions and experiences in participants’ lives, before process (C) |
| Illustration | At that time many customers bought our yellow rice at school. But there was a gossip ... that made that people did not buy our rice. But I never give up. ... Even though the food was left over, next day I kept selling the food. The gossip came again, but I kept selling the food. ... I sold my bicycle, my hens, I sold everything but I never give up, sir ... if I had stopped that meant that I had lost. ... Finally, I could sell the food. Things went back to normal. I think if I had given up at that time, I would not have been selling food anymore. (Man, aged 59)  I am the one who feels ashamed. My friends treat me as usual. They do not feel disgusted ... .but I cannot help feeling ashamed. I am afraid they will avoid me. (Woman, aged 43)  When people are gathering and chatting, and I come over, those who do not like me will stride off. (Woman, aged 43)  I felt hopeless. If God had taken my life at that time, I would have accepted it gladly. (Man, aged 39) |
| Finding | Impact on the participants - Stage A: Engaging participants, Increasing individual confidence, capacity and sense of ‘can do’, Establishing inclusive and collaborative group dynamics. (C) |
| Illustration | I became a happy person during that time. people like me do not make things like that. I had a lot of fun. I gained a lot of experience. Really. I could even shoot someone climbing a tree. I was truly happy. (Man, aged 39)  I was happy, but I was also shaking because I had never done that [interviewing a leprosy officer] before ... I was shaking like leaves but happy [laughs] (Woman, aged 26)  I wanted to socialise with my friends [peers]. I wanted to know whether I was the only one suffering from leprosy or if there were others out there. It turned out that I was not the only one who is like this [showing his impaired hand]. (Man, aged 61)  At first, I felt so insecure, but the feeling is gone. ... Maybe it is because ... I also socialise with healthy ... people. ... This handsome young [research assistant] is willing to get along with someone like me [laughs] and ... [this other research assistant] does not mind drinking from the same glass with me. (Man, aged 61) |
| Finding | Stage B: Motivating social dialogue focused on participants’ lives and concerns, Developing critically: group reflection and reframing, Building agency: purpose. (C) |
| Illustration | I learn about perseverance from him [other participant]. Regardless his impaired condition, he keeps up his spirit, and his wife is very supportive. ... It inspires me. He inspires me to do this and that ... He does not feel insecure, neither does he worry that no one will buy his chips. (Man, aged 61)  I want people to know that regardless my imperfect physical state, I do not hide myself and keep doing what I can do. I want that people who watch the video see that people like me cannot do some work, on the contrary, I can do many kinds of work. (Man, aged 61)  I realised that there are many other people, in many areas who suffer from the same disease as I did..... I was touched, and I was determined to share my knowledge about leprosy to these people. ... That was the sole objective in my mind. ... In the past, I did not even go out of my house because every time people saw me, they turned away from me. I felt ashamed of myself. I hope no more people will have such an experience. (Man, aged 39) |
| Finding | Stage C: Group communication action through video production, Social influence: showing the video in wider forums, New or restored social identity. (C) |
| Illustration | This is the first time I delivered a testimony in front of many people. I was rather doubtful at first whether I can do it or not, but after the testimony when people started raising questions, I felt happy. I want to keep giving testimonials. (Man, aged 39)  I know more about leprosy now, and if you are asking me about my insecurity, I think there is only a very small part of it left in me. The knowledge I have recently gained made me more confident. I am positive that I have recovered. (Man, aged 21)  After video activity, I feel full of spirit. When people say something bad, I do not let it bother me...... As long as I do not cause trouble for other people, I have nothing to worry...... I have recovered now, so let people talk! I feel free these days. Nobody stops me from going here and there or from doing this or that. (Woman, aged 26)  I do not feel shy towards my neighbours any more. I chat with my neighbours and friends. Things have gone back to normal. (Woman, aged 26) |
| Finding | Understanding difficulties: Concealment (C) |
| Illustration | I do not mind the video being played anywhere because people have known my real condition. There is nothing I can do about it. However, I am afraid that my husband and other relatives object to the idea. I want people to know about this, but I am worried about my family’s reaction. (Woman, aged 43) |
| Finding | Physical limitations (C) |
| Illustration | My problem is my physical limitation. I felt tired quickly, but thanks to God, I could stay in the process until the end. I was afraid of getting sick because of the activity because I usually get sick when I am too tired. (Man, aged 61)  You see the condition of my arms and hand. It is not easy to shoot with this kind of arm. However, everything is possible if we are willing to learn. I am happy because I finally could do it. (Man, aged 39) |
| Finding | Behaviour caused by internalised stigma (C) |
| Illustration | At first, I felt insecure and ashamed. I cannot tell you how, but that was what I felt ... After I knew the activity better, I felt comfortable doing it. I could meet many people and we could share with each other. (Man, aged 21) |
| Study: Leprosy, the key to another kingdom 2011 [31] | |
| Finding | Stigma in and around the leprosy colony (N) |
| Illustration | Leprosy-affected people in the colony were forced to leave their families due to their disease, and also because of reduced marriage opportunities for their relatives. Similarly, leprosy-affected people living in the adjacent neighbourhoods had difficulties to find a marriage partner. |
| Finding | Origin of the colony (C) |
| Illustration | The hospital was built at this particular place because there is a hill. Inside the hill, there is some remedy for this kind of disease. The hill was perceived as an outstanding geographic characteristic of the area and served to emphasise the differentness of the people who lived there. |
| Finding | Community membership: The concept of a collective leprosy-affected body, Stigma as a means of fundraising, Distinctive social values within the colony, Control over community membership, Leprosy versus colony member identity (C) |
| Illustration | Who do you mean? We have all leprosy! It is a disease through which the whole family gets neglected. We have the same trouble as our parents, although we are healthy children. Because of our deformities caused by the disease we can’t work. We can’t do other work than begging. […] We are also unable to do work like teaching, which can be done with physical deformities. The reason is that none of the affected people is educated.  In earlier days, when our disease started, we were neglected and ill-treated. And we were also not allowed to acquire good education. I have a very happy life here. I don’t feel like going back to my relatives because they neglected me when I was a child.  The people in the colony are more important to me than anybody else. ...... I don’t care for castes or religiousness. The colony is our kingdom. Here we are the kings. The money matters there [in the colony]. They want to earn somehow. Even begging is one of their sources...... |
| Study: Disclosure of Disease among Women affected by Leprosy: A Qualitative Study 2020 [32] | |
| Finding | Fear of the disease (C) |
| Illustration | I feel afraid if my friends come to know, they will gossip about my health condition and ignore me; also, I lack interest in my studies. |
| Finding | Positive experience after disclosure (C) |
| Illustration | In my village, all the community members are aware that I have been treated for leprosy, but still there wasn't any problem with me. I as usual participate in all the social activities and my neighbours come to my home just like before.  In my village all of them know I was affected by leprosy, and we are all living together without any problem. Due to ulcer on my foot, most of my neighbours know I was affected by leprosy, but they never neglected me. I myself feel inferior and avoid participating in social activities and functions in my village. |
| Finding | Violent reaction after disclosure (C) |
| Illustration | My husband called me as a leper and abused me whenever he had alcohol. He even rejected and did not support me get treatment in the early disease period (early stages of the disease). |
| Finding | Psychological issues (C) |
| Illustration | I was very upset and depressed and had suicidal thoughts many times due to this disease.  Nowadays I get unnecessarily stressed and afraid to tell anyone. Due to the visibility of patches on my face and hand, all of them came to know I was affected with leprosy. But myself getting angry and frustrated to live, asked myself, Why me? What sin have I committed? |
| Finding | Self-stigma (C) |
| Illustration | By God’s grace, I have only patches in my body which are not visible to others. If I had any signs and physical deformities, people may easily identify and discriminate me. |
| Finding | Problems in practicing self-care (N) |
| Illustration | avoided using micro-cellular rubber (MCR) footwear in her village due to fear of stigma and discrimination. |
| Finding | Stigmatisation and family members (N) |
| Illustration | Fear of disclosure of the disease status to husband and in-laws due to fear of divorce. |
| Finding | Beliefs and myths attached to leprosy (N) |
| Illustration | Fear her enemies would send evil spirits towards her and her family if they knew she had been affected by leprosy. |
| Finding | Experience of societal stigma (C) |
| Illustration | When I started to come to the hospital for leprosy care, I came to know that there are some people from my village who are already affected with leprosy, and so the community members have separated and sent them to the nearby leprosy colonies, so I started to hide about my health condition to my husband and my in-laws.  Only my husband knows about my disease status and I fear to disclose it to my remaining family members, because I had an experience in my village about 10 years ago. There was an old lady diagnosed with leprosy, so the community members isolated her in a nearby farm; after a few months the lady died, without any help from others. |
| Finding | Employment issues (N) |
| Illustration | Feared disqualification from her post as a Panchayat member in her village if community members found out she had been affected by leprosy. |
| Study: The way women experience disabilities and especially disabilities related to leprosy in rural areas in south Sulawesi, Indonesia 2010 [33] | |
| Finding | Work (C) |
| Illustration | If I was healthy I would get a job easily, especially with my educational background. But with my condition, of course it is different, because I am disabled.  My children usually warn me. They say: ‘Come on mother, you don’t need to do that.’ For instance when I try to lift heavy things. When they say that, then I’ll not continue that, I just leave it. My movement is limited and sometimes I do not feel well. Heavy things that I grab usually fall out of my hands.  I can’t earn money for a living. Only if I go to my nephew’s house he usually gives me money like 20 thousand, 30 thousand or 50 thousand Rupiah (about €2, €3 or €5). |
| Finding | Social activities (C) |
| Illustration | When there is a community gathering in the public affair office, I’m not invited. I do go to the Maulid (Prophet Muhammed anniversary), which is celebrated in the mosque. Usually my niece picks me up. Right now, it is far different than when I was healthy. In former times, when there was a family having a celebration of an event, I always came if they invited me, although it was far away. Now not anymore, except for them who live very close. Then I still come, if they are close relatives and living nearby. |
| Finding | Acceptance by the community (C) |
| Illustration | Healthy people seem to be very sorry for us. But sometimes their deep compassion creates a different treatment. What I mean is, of course their behaviour toward us and toward the healthy people is not similar. We have to understand that. Actually, I feel embarrassed if people see my condition like this. If possible, I do not want to see them. I have no friends. No boy will ever want me. |
| Finding | Key-informants: Knowledge about leprosy, acceptance by the community, marriage prospects (C) |
| Illustration | The cause is curse or spells. If it is a curse, there is no cure for the disease. The person who has it and his / her family will feel very humiliated. It is different if it is caused by spells. People do not feel that embarrassed because it is a spell and it is curable if you bring him / her to a ‘smart healer’. As a matter of fact, there’s no problem when they are cured. But even then, many people feel disgust even though they are cured. That’s because the signs they have, like mutilated fingers.  It is also difficult for someone to get married, when people already know that he/she has a family member suffering from leprosy. Other people will be afraid of getting the disease, so they refuse to propose to a family member of that sick person. |
| Study: From contagious to chronic: A life course experience with leprosy in Taiwanese women 2006 [34] | |
| Finding | Before being diagnosed: Aware of being different in the early stage of disease but did not know the cause of problem, Symptoms would not go away (N) |
| Illustration | They noticed symptoms and changes in their face, skin, hands, fingers, and feet during childhood or adolescence. They suffer from burns, ulcers in the feet, and skin rash. They had no idea what caused the symptoms, often choosing to ignore the symptoms and hoping that they would go away. Diagnosis was delayed because of poor knowledge - they tried home or folk remedies, avoided certain foods, or used herbs. After these remedies had failed, they would seek help from spiritual gods. Many general practice doctors misdiagnose them as they were not attuned to leprosy diagnosis and treatment. |
| Finding | After being diagnosed: feeling of shame and being stigmatised, conflict about treatment at Institution, motivated for treatment to be leprosy-free (N) |
| Illustration | These women stayed in the house as much as possible, covered their arms and hands, stopped going to school, avoided being seen when someone came to their house, and paid few visits to relatives and friends. They were subjected to intentional or unintentional stigmatising behaviours by others and several considered suicide. Seeking admission to treatment facilities either by being forced or voluntarily had its benefits and problems but these women were motivated to receive treatment and be leprosy-free so that they can return to their families. |
| Finding | Living with leprosy: Missing early attachment to own children, Loving, protecting and being proud of offspring, Striving for family, social and religious support, being as useful and independent, (N) |
| Illustration | These women who were separated from their babies or who had to avoid nursing them for fear of transmitting the infection were filled with guilt and sorrow at missing their early years. These women were careful to make sure their children did not get leprosy and were grateful that they turned out healthy. They were severely limited to assist them in advancing their education and career but supported them with their love and were especially proud of their success. They were able to reconnect with their families and reconstruct a web of social support and religion played a big role in their adjustment to leprosy and its complications. Despite their various degrees of disabilities, most women managed their own daily needs, helped care for their grandchildren, did some work in exchange for cash, ran errands, volunteered or planted flowers and vegetables. |
| Finding | The future: Once leprosy, forever leprosy, Preferred to stay in the leprosarium and LLTCC than move to the community, life is more difficult with pain, disabilities and chronic diseases, The final days (N) |
| Illustration | Despite being disease-free, these women still bear the physical changes (disabilities and deformities) of leprosy, and they interfered with daily routines and socialisation. The periodic phone calls from the nurse for follow-ups remind them of leprosy. Many of them who have lived at the leprosarium for >10 years did not want to leave because they were afraid the community would not accept them, they had no families to return to or they did not want to burden their children, they were not confident to live independently, and they would not be able to afford the expenses. The women in their 70's spoke freely about death especially when the nerve pain was severe and they were grateful to God for allowing them to live this long despite their physical limitation |
| Study: A qualitative study exploring the perceived impact of race on leprosy-affected persons' experiences of diagnosis and treatment of leprosy in southeast Brazil 2021 [35] | |
| Finding | Racism is part of Brazilian culture: Blacks are the most discriminated race in Brazil, No direct role of race in leprosy treatment (C) |
| Illustration | I listen to people talking on the streets, 'look at that nigger!" ...Even Japanese people suffer from discrimination. (P89, Brown)  It ́s when those people, because they are white, come to me and say: “Hey Nigger” (P44, Black)  Because of slavery, they are considered an inferior race. (P85, White)  We see that in the houses of the white people, they fear us more than the other races. (P100, Black) Because you are an Indian or black you don't have rights...I feel sad because I feel discriminated. (P14, Indigenous)  Where I attend, all coloured people, darker, whiter than me, even red faced, are treated in the same way. (P85, White)  I've seen this here in the hospital...some discrimination towards black people. (P99, White) Here [in ILSL] I've never experienced racism, but at the health centres I have. (P1, Brown) |
| Finding | Difficulties associated with leprosy diagnosis: Stigma and discrimination, Lack of knowledge (C) |
| Illustration | Because everybody knew I had leprosy, all my friends stayed away from me. They were afraid to get it. I had a boyfriend and he left me too. (P1, Brown)  When I left the doctor's office the nurse told me not to tell anyone I have leprosy. Because of discrimination. (P60, Brown)  Sometimes we discriminate ourselves... I don't offer coffee to somebody in my house because I don't know what she's going to think about the water I serve, so I don't serve anything. (P3, Brown)  I only got information after leaving here [ILSL]... I didn't know so I expected that my arms and hands would fall off. Nobody would tell me anything or give me any information. (P99, White)  How did I get this disease...? In the paper it says that it comes from an armadillo ...but I have never eaten an armadillo? (P30, Black)  I want to know where leprosy comes from because I've never had contact with a person that had it. How did I get it? (P85, White)  I asked myself when would I die? I sat down on my bed waiting for death to come. (P99, White) |
| Finding | Barriers to accessing treatment: Lack of adequate treatment centres, Stigma from healthcare professionals (C) |
| Illustration | I used to live in [a small city] ...I was there for five years but they didn't know what it was. I couldn't even walk and was in a lot of pain for five years. Only after a biopsy in [a bigger city] they found out. (P1, Brown)  I had two or three wrong diagnoses until I got someone who referred me to come here to ILSL. (P100, Black)  I would have done anything to get the treatment...It took about two years until I was referred here and got the right treatment. (P39, White)  I've had leprosy for more than 17 years. After that I couldn't work anymore. So I started receiving a pension from the government. We suffer and don't have the money to buy medicines. (P14, Indigenous) They don't give me the prescription where I live, I have to come here [ILSL]. It is difficult to get transport. (P100, Black)  Where I live is very far from here and I have no money to get here. (P60, Brown)  Some health professionals were afraid to treat patients because they thought they could get the disease. (P7, White) |
| Finding | Lack of health education: Education through schools, Improve health education strategies (C) |
| Illustration | It is like super-glue, that sticks [discrimination] and doesn't come off anymore. Even us, ourselves, we have some kind of prejudice. It is cultural. It is hard. (P100, Black)  I think they should do more than just talk. They speak about leprosy on the radio and nobody cares because they don't know how to understand it [lack of education of population]. The lack of education is the problem. (P99, White) |
| Study: A temporal and sociocultural exploration of the stigma experiences of leprosy patients in Brazil 2016 [36] | |
| Finding | Changing attitudes towards leprosy patients in Brazil (C) |
| Illustration | The doctor said I had to go to the hospital. That I had to burn all my clothes that I used, to throw away all the dishes I used, separate the plates, spoons I had used, things like that. (P9, 56 year old female, diagnosed in 1973)  [People] had prejudice:::it really was ugly:::We were treated as a mangy dog. [We were]:::totally excluded from the society. (P19, 81 year old female, diagnosed in 1943)  The treatment has changed; the physicians studied more about it:::They said No, it has no danger:::there’s no need to have prejudice. (P24, 70 year old male, diagnosed in 1973)  I believe it is more normal. Because there is the vaccine so you don’t contract it. (P26, 16 year old female, diagnosed in 2014) |
| Finding | Acts of discrimination experienced by leprosy patients - By the government, family members, workplace (C) |
| Illustration | It was a compulsory hospital admission. I didn’t want to, I was forced to come. (P14, 83 year old male, diagnosed in 1957)  I had a newborn, I think she was not three months, and then I had to leave [and go to the hospital]. Yes, to leave a child while breastfeeding right? It was not easy. (P8, 56 year old female, diagnosed in 1984) [My wife and I] sleep apart from each other because of this problem of mine. Because this disease transmits to another person right, so she didn’t want to be next to me. (P5, 57 year old male, diagnosed in 1992)  My own blood:::If I go in my mother-in-law’s house and she offers water and I drink:::She throws the glass away....They have a huge fear of catching [leprosy]. (P7, 21 year old female, diagnosed in 2013)  The director of the school wanted me to ask [for] resignation. I worked there for many years...... I didn’t believe in prejudice until I faced it. And it was really hard, traumatic. .....They damaged my mind.... (P17, 51 year old female, diagnosed in 2009) |
| Finding | Complications of disability: Experienced stigma, Inability to work and loss of social participation (C) |
| Illustration | Ah, I don’t know:::when you have a crooked hand, fingers, a deformed leg, these things. People change with you a bit:::well the prejudice happens:::they kind of get away from it. (P4, 67 year old male) Previously I did everything. I used to work, I used to take care of my mother :::[but] it changed now, now that I hurt my foot, I cannot walk. I get kind of angry, because I cannot go anywhere. (P6, 68 year old female)  A person invites me to go out on Sunday, calls me to go to the ranch, to go to mass. I used to go every Sunday and I’m not going anymore. (P23, 54 year old male)  My husband made me quit. Because I had pain. Because I got neuritis. And with no study, the only work that was left was as a maid. And I couldn’t do that [either]. (P10, 51 years old)  [It felt like] everyone was against me.... I blamed myself. I noticed that I was getting depressed. I didn’t want to go outside. I didn’t want to have contact with anyone. (P7, 21 year old female)  ..... I’m ashamed [of myself]. (P8, 56 years old) |
| Study: Leprosy as a neglected disease and its stigma in the northeast of Brazil 2014 [37] | |
| Finding | The changes that occurred in the family after the diagnosis of Hansen's disease: religiosity, prejudice and aversion (C) |
| Illustration | My family was very shocked. However, today they are used to it. Every day my mother says some prayers. She says these prayers for me and I accept them. (P 4)  No. Only my husband teases me once in a while. He calls me a leper. When he is angry he does this. (P 14)  Regarding the people in my house, it has been a bit weird. Although they haven't kept away from me, I noticed certain changes. For example, they don't eat off the same dish. |
| Finding | Neighbours' and co-workers' attitude in the face of the diagnosis of Hansen's disease (C) |
| Illustration | My colleagues - most of them don't know. I am not going to tell, am I ? Some colleagues who know about the disease don't bother, but others keep a little distance. They don't come close to us. I feel some rejection already. (P 3)  Well, I wasn't so well-known on the street, but after I caught this disease everybody started to stare at me...but I don't take it seriously, you know? (P 6) |
| Finding | The consequences in the patient's social life (C) |
| Illustration | After these problems started to appear in my skin, I started to spend less time outside. (P 1)  My life stayed this way, the folks stayed away from me. We can see this. Some of them completely stopped going to my house. Only some of them keep on going there. (P 2) |
| Study: PERCEPTION OF PATIENTS WITH LEPRA ABOUT THE SELF-CARE GROUPS 2018 [38] | |
| Finding | Importance of self-care for the prevention of physical and psychosocial disabilities (C) |
| Illustration | Always take the medicine right [...], wash your feet [...], take care ..., give moisturizing oil [...]. Self-care all [...]. Sometimes we do not do everything completely, because it's a lot for us to do [...]. When standing in front of the television, put your feet in the water for ten minutes [...] and then [...] hydrate [...]. Then we feel better. (page 04)  Look, I learned to take care of the eyes, to take care of the nose, right? [...]. Up to your ear, eh? And the very voice, which is [...] the throat of the people, is a little dry. And we must also be careful, be guided and [...] heard as well as members, right? Nerves, arms, feet ... these things [...]. All this we could handle better, right?. (p. 06)  When I come here, I feel better and we talk, try to know things. There was one time that I wanted to stop taking the medicine, because I take medicine and never get well. I kept thinking: no, it's better to do what has to be right. (page 07) |
| Finding | Contributions of the SSG in coping with the difficulties and limitations of people affected by leprosy (C) |
| Illustration | I was crying in the room [...]. You do not know how hard it is to get here. And the way she [healthcare professional] welcomed the patients hugging, kissing, when she gave me the first hug I thought: she kissed me! I was horrified! This woman must be crazy or she must have the disease right? (p. 11)  At first there was a lot of prejudice, but after I joined the group, we made new friends. That attracted a lot of good things, because before I felt everything out there [...]. After the group, I accepted the disease better. (p. 05)  I lived very depressed [...], I lived thinking. I said, my God, take care of me, Lord [...]. But then I'm glad to be coming here to the group. (page 01)  It has changed in my life that sometimes, when I come here myself, I feel better. We talk and look for things. (page 07)  Just the fact of coming and participating in the group, seeing that there were people with more difficulties than I, with more weaknesses that I had and was there strong and strong, why I could not be? (p. 11) |
| Study: The experiences of people affected by leprosy who participated in self-care groups in the community: A qualitative study in Indonesia 2017 [39] | |
| Finding | Self-perceived condition: Understanding the disease, Self-image related to their disease (C) |
| Illustration | Actually the leprosy is a little wound that is not considered serious but it gets worse. However if it is treated, it will not be contagious and if the treatment is late it can be contagious. A disease like this should be treated fast, with the hope of recovery, but if not handled properly, it will be contagious, because the disease is indeed a contagious disease and without fast treatment it will continue to spread continuously throughout the body leading to injuries and disability.  The first time I felt and saw the sign, I thought it was an allergy. My skin disease is basically only a skin disease, and I do not know if this is leprosy and if it is contagious to others. Let others say anything they want, I do not care, but I am a breadwinner for my family, so I have to keep working to meet all the needs of my family. When people knew I got leprosy, I felt those people were too quick to stay away from me:::  I isolated myself at home after the leprosy was detected for three months due to shame. |
| Finding | Adherence to treatment: Lack of confidence in treatment by PHC, Understanding MDT regimens, Reducing side-effects of treatment (C) |
| Illustration | We were taught by public health nurses (PHNs) about leprosy treatment and how to follow leprosy medication related to the time of taking the medicine, regularity and the positive impact of leprosy treatment received every month directly from PHCs.  I took medication for the one-year programme, but I stopped for one month because I was lazy and wondered why it was taking so long with no result, so I had to repeat again for one more year because it was declared zero again by the officer.  We were also worried because after taking the medicine there were some complaints like blackened skin, boils and blisters on the skin, red urine, and lost appetite. The PHNs explained that they were all normal side effects, so we were asked to continue treatment and we were given additional drugs to reduce the side effects. |
| Finding | Ability to do self-care: Basic human needs, Control living environment, Use of personal, protective equipment, Skin and wound care and prevention of disability, Participation in self-care group (C) |
| Illustration | ... We were asked to eat nutritious foods; ..... and mostly consume tofu and tempeh. I have to take a shower twice a day .... The home should have plenty of ventilation and sunlight, we should clean the house often.... PHNs give counseling to protect patients with personal protective equipment because the eyes, hands and feet are very sensitive. ..... It is always repeated how to care for hands and feet that are numb or wounded, to prevent disability.....  At this self-care forum, we discuss various problems experienced by people suffering from leprosy..... Sometimes we are given some entertainment to reduce the stress we feel! We are also given a self-gym for exercise to reduce disability and the peripheral nerve damage. |
| Finding | The kind of help and services received: Those based on ancestral cultural/religious heritage, Traditional and alternative medicine, Modern services from health workers (C) |
| Illustration | I did a lot of efforts to eliminate this disease..... I went to a healer to seek treatment and was given prayers, amulets, and potions but it did not work and the disease only got worse.  I bathed in sulfur water on the mountain but ....there were also cuts on the soles of my feet and fingers and I compressed them with cassava ... but instead, the wound rotted and eventually I got my legs amputated.  I went to the doctor and the doctor said this was .... leprosy and he ...asked me to come to PHC.... to get free treatment. So I went to the PHCs and got this MDT package every month, initially I was on 6 months treatment and moved to 12 months treatment...... I took a break from the drug and I got great side effects.....so I was referred to a leprosy special hospital and treated for a month and there were some of my fingers broken off because of my own defects and my nose is twisted and my ears are tilted and curved. |
| Finding | Acceptance and support for leprosy patients: Family support during treatment, Public social acceptance toward leprosy clients, Provision of adequate information and health services from public health centers, Return to work and acceptance at work after recovery (C) |
| Illustration | The family wanted the best treatment for my illness and motivated me to strongly undergo this treatment because my neighbours can also recover without any symptoms remaining, as long as the patient diligently undergoes treatment at the PHCs as directed from PHNs. Every Saturday in the first week of every month, PHNs at PHCs always guide and coach us about self-care in SCGs forum. Infrequently, health education is also provided on the latest rubbing movements, MDT treatment guidelines and training for leprosy patients. At this self-care forum, we are also taught skills such as sewing or carpentry or making crafts in order to be able to work. We’re also taught to train the movement of the finger so it did not go paralysed. Unfortunately, there was no follow up after the skills were given, especially on how we can start a business or get back to work. |
| Study: Leprosy in kiribati: The lived experience 2020 [40] | |
| Finding | Recognising leprosy (C) |
| Illustration | It happened while I was in primary [school] ... it occurred on my leg and arm ... just a dot ... just a white patch ... (Young woman, 20s)  It’s very scary this disease known as leprosy ... It’s quite quick to spoil the body of affected people during that time ... Just like it’s no longer the face of humans ...’ (Older man, 60s)  Now we know that leprosy can be transmitted ... we no longer believe it’s in the blood line ... the disease can be transmitted ... Also, it can be cured with available treatment (Mother, 30s, who attended the interview with Young man, teens).  When I listened to the radio and I knew it’s leprosy ...from my hand ... and I began to feel frightened ... Mrs [to wife], you got money (why?) ... it’s better that I go to Nawerewere and find out exactly at the leprosy office... (Older man, 60s).  I didn’t know she was sick ... such that when I bring her fish for food and I will often lie down ... even beside her on her sleeping mat... (Older man, 60s) |
| Finding | Stigma (C) |
| Illustration | I didn’t want to come here (hospital) ... because I’m ashamed (Young man, teens) ... because I was ashamed ... I really don’t want to be a leper...’ (Young man, 20s) My brothers were very surprised ... ‘how come he got the disease? Are you treacherous?’ ... I was quite saddened ...sometimes they look at me ... their eyes – were unhappy (Young man, 20s)  ... we have to distance ourselves for a while ... my grandmother told me to keep at a distance [during sleeping time] (Young woman, teens) ... he spoke – and kept referring back to my illness such that I would feel subdued by it: ‘you are a leper and a sick one’ ... it became his golden verse ... when he becomes angry he blurts it out ... (Young woman, 20s)  ... I went to school and they [classmates] asked, ‘What happened to your eyebrow?’ And I replied that I shaved it ... (Young woman, teens).  First she told me – ‘you look dirty – go wash your face ... it’s really darkened’ ... and then I wouldn’t want to attend school (Young woman, teens). |
| Study: Gendered experiences: marriage and the stigma of leprosy 2006 [41] | |
| Finding | Stigma: Perceptions and Behaviour (C) |
| Illustration | Usually when they [bathers at the river] saw me, they put their hands on their face [because] they thought that if they didn't do like that the disease would be transmitted to them. They were thinking leprosy was very infectious disease, it could transmit through contact, through air, or faeces. (Male 15) even the sweat transmits the disease (Female 4).  ...it is transmitted through air, urine and defecation. So, if you work with us we could get this disease (Male 17).  I had been asking with God that what did I do so he gave me this type of punishment (Male 15).  They both get equal treatment. They force them to live an isolated life on the bank of some river or a solitary place. ..... If they have leprosy, they get equal treatment (Male 1).  My villagers told me that I could not stay in village. I had to stay in outside of my village. (Male 13)  I was not allowed walking through main road. So usually I came to my house from cottage through side road and after taking food I returned through same road. (Male 15). |
| Finding | Marriage (C) |
| Illustration | All arrangements for my marriage had been done in the village where I used to work. Then...the whole village came to know that I had leprosy. So, the marriage could not take place. (Male 3).  Female will be faced more difficulties than male. Because feeling of male and female is not equal. Male is more selfish than female, they think that if wife will get leprosy then he could be get another wife easily, but female can not get another husband easily. Yes male can earn and there is much option for them but for female after marriage there is no other choice. (Female 13)  Even if I like someone he will not accept me because I am a leprosy-affected person. (Female 19) ...because it might cause problem in the marriage of [his] brothers and sisters. It hurt me so I left. (Male 3)  In Terai if you are from leprosy-affected family marriage won't happen and if they get married then lots of dowry will be asked for. (Female 2) |
| Finding | Attitudes and perceptions (C) |
| Illustration | “I am not capable of doing anything", "Do you think I am worth doing something?” (Female 8) |
| Study: The impact of leprosy, podoconiosis and lymphatic filariasis on family quality of life: A qualitative study in Northwest Ethiopia 2020 [42] | |
| Finding | Physical: symptoms, cause and self-care (C) |
| Illustration | ....First it started when I swam in the river with a scabies-like rash on my whole body and it was itching, finally the wound started from my foot and spread to my whole body, then a feeling of senseless, finally it eats my fingers and I lose my fingers. . .” (Man with leprosy, age 60)  . . .My father and I assumed that the disease would be transmitted to my children but the reality is not that because my children are still not affected now. . .” (Man with leprosy, age 64, FGD) |
| Finding | Psychological aspects and mental wellbeing (N) |
| Illustration | Some participants said that they felt inferior compared to their friends or community members. ...one person affected by leprosy from the focus group discussion said he used to have suicidal thoughts. |
| Finding | Level of Independence: Day-to-day life, work and resources (C) |
| Illustration | . . .I work in handicraft since my hand’s fingers are well (. . .) I cannot do my previous agricultural work because of my disease. If [I’m] exposed to soil and mud my wound aggravates, that makes me poor. I thank God my hand is well . . . (Woman affected by leprosy, age 56) |
| Finding | Environment: Attitudes and social participation (C) |
| Illustration | . . .Many of my neighbours used to say we cannot enter their house, they separate me from coffee [ceremonies] too. Sometimes when I said hello to kids their parents were not happy, some of them warned me not to touch them. That was the worst time during my illness (. . .) When people discussed my disease and prevented [me] from social life my wife asked me to divorce. . . (Man with leprosy, age 45) |
| Finding | Social support and family relations (C) |
| Illustration | ....My wife repeatedly asks me to be divorce and even she was lost for more than two weeks. Then I begged the elders and priests in the town for her to come back. Especially her relatives forced her to leave me . . .” (Man with leprosy, age 45)  “. . .[I] divorced with my husband (. . .) He married another wife and had two additional children (. . .) It was the worst situation in the last times to live with the community but now it is improved. I feel ashamed when people [include] me in social interactions. . .” (Woman with leprosy, age 56)  ....My home renter told me to leave his house since I cannot pay on time. I was forced to leave his house with my children. However, my neighbours pay my rent (. . .) my neighbours lend me money for holy water and other expenses (. . .) The people around me helped me what they can . . . (Man with leprosy, age 38) |
| Study: The Impact of Leprosy on Marital Relationships and Sexual Health among Married Women in Eastern Nepal 2016 [43] | |
| Finding | Leprosy-affected women (C) |
| Illustration | ...Before there were problems, I did not give them [family] food which I had taken. I was worried that it would transfer to them.... (Woman with leprosy, age 33)  ...My husband is afraid that it transmits through respiration, so he does not want to tongue kiss for seven months....(Woman with leprosy, age 22) |
| Finding | Marriage, Sexual relationships, Sex Education (C) |
| Illustration | ...Yes, it is also important for me, but our importance has no value. We cannot express our feelings even with our husband.... (Woman with leprosy, age 26)  ...It is not necessary how important it is for us because whenever our husband is ready we should be ready.... (Woman with leprosy, age 50) |
| Finding | Factors affecting the marital relationship of women (C) |
| Illustration | ...The most important is trust, love and understanding.... (Woman with leprosy, age 32)  ...When I was diagnosed with leprosy I felt that my husband’s behaviour had changed, he did not share anything with me and he pretended to be busy with work. But actually he was trying to be far away from me.... (Woman with leprosy, age 33)  ...Before there were problems, when my father and mother-in-law knew about my disease, they hesitated to talk to me and come near me.... (Woman with leprosy, age 33) |
| Finding | Factors Affecting the Sexual Relationship of Married Women in Nepal, Positive and negative factors (C) |
| Illustration | ...Love helps for the good sexual relationship with my husband.... (Woman with leprosy, age 35)  ...He has the bad habit of drinking alcohol. He wants every time when he is drunk. I feel so irritated but what can I do, we think of our husband as God and we should obey him....(Woman with leprosy, age 26) ...When I do not want to have sexual intercourse, my husband forces me. He scolds me “I used to earn money, bring food for you all but you do not want [to have sex], then get out of the house!” Sometimes he raised a hand on me. So I have to be near and close and have sex with him.... (Woman with leprosy, age 32)  ...At first when he knew that I was affected by leprosy he did not sleep with me.....he asked the doctor about the sexual relationship. He was told that it does not transfer to him so he started having sexual intercourse with me again.... (Woman with leprosy, age 32)  ....I do not want to remember the past and talk about that.... (Woman with leprosy, age 35) |
| Study: Illness perceptions of leprosy-cured individuals in Surinam with residual disfigurements – “I am cured, but still I am ill” 2017 [44] | |
| Finding | Illness perceptions: family curse, food and heredity (C) |
| Illustration | It is hard to understand these things (possible causes), you will not understand but in Surinam we believe that when people do bad things...this will result in having deficient children. For example, my grandfather had kicked a boa constrictor to death, and my mother had beaten a fat frog to death. There were a thousand and one ‘treven’.  My diet consisted of a little bit of rice and dry fish. And so I grew weak. I lacked resistance, and became skinny and weak. When I came here (leprosarium), you know who were here too? My uncle and a cousin. |
| Finding | Concealment (C) |
| Illustration | I am cured, but still I am ill. When they do not see it (her hands), they will not look at me... . And when people persistently stare at you, you become, as being a human, nervous and shy. To prevent this, I always do like this’’ (putting her hands under the cloth). |
| Study: Mental wellbeing among people affected by leprosy in the Terai region, Nepal 2021 [45] | |
| Finding | Disability grade (C) |
| Illustration | ‘Before I was diagnosed with leprosy, I did all types of work. I brought food for my husband to Janakpur. I collected firewood and dried leaves. Now I am unable to do any work. I cannot see anymore. I am unhappy.’ (Female, over 55 years of age, DG 2) |
| Finding | People’s feelings and experiences regarding leprosy (C) |
| Illustration | Yes, I feel ashamed due to my disease. Even if you are sitting in front of me, I feel shameful. How could I sit in front of you with my disease? ... Some people said: “Aunty why you cover your whole body with clothes and sit separately? I said I have a disease; it hurts me and makes me shameful”.’ (Female, age above 55, DG 2)  I felt ashamed, that’s why I did not tell them (family), I was worried about my disease. When I started to take medicines then they (family) knew about my disease. (Male, age 60, DG 0)  No, I don’t feel afraid if people know about my disease. I walk freely, I am fine. (Male, age 43, DG 1)  Now there is no problem but before, 7 years ago, when I married off my daughter. The family of the groom did not accept my daughter due to my leprosy. They told me that she is the daughter of a leprosy-affected person. I went to the police station and won the argument. (Male, age 47, DG 2) |
| Finding | Social and daily life factors: Family and community, Work (C) |
| Illustration | Yes, sometimes I felt I was losing my self-respect due to this disease because some community people teased me while talking sometimes. I felt let down by my family. My family never cared for me. (Female, age 60, DG 1)  No, I felt less [unsatisfied and sad] at that time of diagnosing but my son encouraged me. He said: “it’s nothing, do not worry, now you start medicine, I do not mind.” ... Then what can I say. (Male, age 60, DG 1)  I’m not worried about my disease. I am worried about my foot. Without it, I could not work like other normal people. I feel jealous when I see other normal people.’ (Female, age 60, unemployed, DG 2)  Every time I stay at home. I am not able to earn for us. There are 5–6 members in my family who need food and only one person who earns for us.....(Female, age 35, DG 1)  I felt pain while doing my work. I felt weakness in my hands and legs, both. I cannot do hard work. I have to pay more money to the porter who manages all the food while I do my business. (Male, age 60, DG 2) |
| Finding | Cultural factors (C) |
| Illustration | .....Now, I have taken medicines from Lalgadh. I believe that the disease cannot harm me anymore [...] If we do regular activity (soaking, self-care and medication), we will be safe from the bad effect of leprosy. Otherwise, it’s harmful to us. (Male, age 60, DG 1)  I suggest to people it’s just a disease, you must go for treatment on time, otherwise it will infect other people in your family to. It will make you disabled. If your family gets the same symptoms send them to Lalgadh as soon as possible.’ (Male, age 60, DG 1)  When my husband brought me to Kathmandu for treatment, he said to me: ..... if I will die before you, you have to face so many problems, your condition will be just like a dog... (Female, age 60, DG 1)  No, I did not talk with anyone.... I should die, everyone should die one day... God made me like this. I am still crying day and night. (Female, Muslim, above 55 years old, DG 2)  God gave me this, so why should I feel bad about leprosy, I don’t feel shy. (Female, Hindu, 59 years old, DG 1) |
| Finding | Self-help group (C) |
| Illustration | We participate in the SHG. We discuss about saving money and investments, we discuss how we should operate our group smoothly in the coming days. I really enjoy participating in the SHG. (Male, age 60, DG 1)  In the beginning there were only four, five people in the SHG. I asked the doctor for the information about other people who took medicines for leprosy and convinced them to participate in the SHG. (Male, age 60, DG 1)  ‘The facilitator of our group talked and convinced my neighbor/relative who treated me badly, to stop teasing me because of the leprosy. After that she never teased us again. The neighbor also joined the group and know understands more about leprosy.’ (Female, age 35, DG 1).  ‘There are so many people who participate in SHG. They sit together and talk together; I like to sit amongst them.’ (Female, age 70, DG 2) |
| Study: Leprosy perceptions and knowledge in endemic districts in india and indonesia: Differences and commonalities 2021. [46] | |
| Finding | Leprosy perceptions and knowledge in endemic districts in india and indonesia: Differences and commonalities (C) |
| Illustration | . . .[Being affected by leprosy] may be because of wearing wet clothes or some kind of allergy in my blood. It may also be that it had happened to some friend and I got infected while playing because it can spread through the touch. . .–Person affected, male, India, in-depth interview  . . .To my knowledge, women in their menstrual cycle are not permitted to have sexual relations in any religion. So the majority of the community here believe that to be the cause. Bacteria is the cause, and it is believed that [babies] born carrying contaminated bacteria end up having leprosy. . .- male, Indonesia, in-depth interview  . . .When .....[we] were playing on the graveyard, cemetery, and [we] stepped on hot soil. ..... the graveyard is from people with leprosy, so they get infected [by stepping on that land] (. . .) The one that stepped on it is the one that untreatable. But the other friend got it from hereditary. . . male, Indonesia, in-depth interview... |
| Finding | Differences and commonalities in leprosy-related stigma. (C) |
| Illustration | . . .I feel sorry for them. One, because they are alienated from their community. Two, because rarely ever would anyone talk to them or involve them or allow them to raise their own children.... As a community, if someone has leprosy, we fear for our own health, fear of infection. It is horrifying. . .”— female, Indonesia, IDI  . . .Most leprosy patients are dirty and poor. So, if they don’t treat their leprosy it will go on.... If it gets worse, working is not possible....No one would want to work with leprosy patients, no one would like to have leprosy patients employed, no one would buy from leprosy patients. So, if leprosy is visible, they can’t do work - female, India, IDI  . . .People do not eat with leprosy patient nor touch them. And his living place is also separated....Because of this he becomes sad and suffers from inferiority complex. Sometimes he also tried to commit suicide. . gender unknown, India, FGD |
| Study: Experience of persons affected by leprosy in facing psychosocial problems: A qualitative method 2020 [47] | |
| Finding | Anxiety: Symptoms of anxiety, Intensity of anxiety (C) |
| Illustration | My family and I are worried (P2)  I always feel scared (P4) (P6)  My mind (P8) I often . . . (P2) (P6) (P8) I always . . . (P4) |
| Finding | Withdrawal: Barriers to social interractions (C) |
| Illustration | I also rarely leave the house, I feel I am not ready to meet other people (P3) (P11) I do not want to be seen by others when sick (P4)  I prefer to stay at home (P9) |
| Finding | Impaired self-conscept: Body image, Pride (C) |
| Illustration | Because of my body (P5) My body is full of disease (P7) Because my body has blackened skin (P10) I feel ashamed to meet new people and neighbours because of my body (P5) (P7) (P10) |
| Study: Living with stigma: Voices from the Cured Lepers' village in Ghana 2019 [48] | |
| Finding | Length of stay in the Cured Lepers’ Village (C) |
| Illustration | I was in my second year in the leprosarium when Nkrumah’s [overthrow]. When Nkrumah was still president, I was in my village. It was about his 4th year of presidency that I came here. We saw everything that went on then, though I had the disease, I didn’t hide myself, I saw everything. - Mama Yaa (PCL)  More people remained here after cure than those who left. Though there are only fifty- seven (57) residents in this village due to inadequate rooms, there are about (raises hand to count) six smaller villages around with cured lepers’ village who also access our resources and facilities. Some of them have died over the years. - Mr. Klo (CLV)  When they were treated and cured, we gave them a waybill or certificate that they are cured and to go home. They went home and .... came back to report that they are sick.....but when we say no, they find their way to settle across the street and behind the leprosarium which was later named Freetown. - Mr. Abaka (Ho Polyclinic) |
| Finding | Isolation, abandonment and neglect (C) |
| Illustration | ......I was sent into the bush to live there and a fence was made around me to avoid any contact with other people... I could have gone back to live with my brother and sisters but in my hometown this sickness disgusts people. They don’t treat people who suffer this disease well and I was scared of being isolated again. - Papa Kofi (PCL)  . ....They made a different house for me to live in. My plates, cup and buckets were different from what the others used..... No one was willing to come near me..... People who died out of the same ordeal were buried at the same place they lived in the middle of the night...wrapped in a mat and buried with everything they used while alive.....- Papa Senyo (PCL).  Most of my colleagues who decided to go back to their hometowns didn’t even live for two months. They died or should I say they were killed?....they can even kill you over food .... We will rather live here and have peace. - Papa Dela (PCL). |
| Finding | Verbal abuse and ridicule (C) |
| Illustration | I was treated and cured but they still call me a leper.... Some people in our communities even describe our children with the disease. ...How can we live among people with such attitude? - Fo Edem (PCL).  I have decided to be here until I die, and I have said that when I die, I should be buried here. I don’t want my corpse to be taken to my hometown because I don’t want to be called a leper even in death because I know that will embarrass my children a lot. Fo Korsi (PCL)  The main reason I did not return home and divorced my husband was because I wanted to live among my likes. I realised I won’t fit in [with] normal people. I knew my life wouldn’t be the same again because I know how people like the blind and deaf are treated in our society and when I am here, nobody differentiates. We all live as one and that makes me feel happy.... Daa Ese (PCL)  ...Here, we eat and do many other things together without anyone treating the other differently. - Daa Afi (PCL). |
| Finding | Self-stigma and shame (C) |
| Illustration | I was also with my husband and children. I opted to divorce him after I came here because I was ashamed of myself and I thought he would want to marry again so I didn’t want to infect him with the disease. - Daa Ese (PCL).  I had this wound .... 2 months before I gave birth to my son who is now in his teens. This makes me ashamed of myself. I can never live among normal people like this. ... - Daa Aku (PCL).  ....I know my family feels nauseous about me....so I decided to live here....if I go back....I will rather be a burden to them. Sis Ese (PCL)  People with leprosy are not allowed to live in my hometown. They perform some rituals and take you into the bush out of the town to live there. That is our culture and I can’t go back to break it. When I tried, I wasn’t welcomed, and I understand because, that’s how things are done. Daa Afi (PCL)  ...There are a lot of rules and regulations which are based on the Bible. I can’t go contrary to the Bible, so it is better I remain here .... Daa Sena (PCL) |
| Study: The effects of the stigma of leprosy on the income generation of leprosy affected people in the Terai area of south east Nepal 2006 [49] | |
| Finding | Stigma and Income loss (C) |
| Illustration | ... no one was ready to give me work. They all hated me and the restaurant where I was working sacked me telling that I had leprosy and this could be transmitted to others if he stays here. I was in complete destitution, without food, water and shelter. No body allowed me to stay at their place. I had to beg alms for food in Dharan... (Male 1)  Yes, this was the only reason, when the customers saw me with leprosy, they stopped coming to the restaurant and the business was almost a flop. So, I was sacked. (Male1)  When after I got serious wound, usually they did not want to give me offer to work. But some times they offered me work when no other labourers were available there. Because there were wounds in my hand and foot they usually told me that I had to work separately, not with them. Other labourers usually told that if I went to anywhere for work they did not want to go there for work (Male 15) |
| Finding | Income loss due to physical effects of leprosy (C) |
| Illustration | My dad is unable to work because he has deformity in one hand and one foot ... (Female 2).  Although we are labourers, due to our physical problem we are not able to work and are sometimes suffering without food for many days (Female 12).  ....... I used to do all kinds of work, household and working in the field. Then I got a wound and then I stopped working (Female 18)  ..... the disease has affected my hands. I cannot hold things properly with my hands. I can do only easy work now. People have no sympathy with me. No one offers me any job. The previous life was undoubtedly better (Male 1).  .....I can't do work in my full capacity. I don't get appropriate diet. Since there is ulcer in my feet, I am unable to work in the field (Male 9).  I was making more income when I did not have leprosy, but after this disease I became weak and my income was poor. Now I am doing business, ..... it is not necessary for me to go in sunlight or field, it can be done in a room (Female 14) |

**References**

1. Abedi H, Javadi A, Naji S. An exploration of health, family and economic experiences of leprosy patients, Iran. Pak J Biol Sci. 2013;16: 927–932. doi:10.3923/pjbs.2013.927.932

2. Araújo de Souza I, Aparecido Ayres J, Meneguin S, Spagnolo RS. Hansen’s disease patients’ perception of self-care from the complexity perspective. Anna Nery School Journal of Nursing / Escola Anna Nery Revista de Enfermagem. 2014;18: 510–514. doi:10.5935/1414-8145.20140072

3. Ayres Jairo Aparecido;Paiva Bianca Sakamoto Ribeiro;Duarte Marli Teresinha Cassamassimo;Berti Heloisa Wey; Leprosy effects on patients’ daily lives: vulnerabili ty and solidarity. 2012;16: 62.

4. Carneiro da Silva RC, AraÃ^o^jo Vieira MC, Mistura C, Olinda de Souza Carvalho e Lira M, Sarmento SS. Stigmata and prejudice: reality of carriers of leprosy in prisional units. Revista de Pesquisa: Cuidado e Fundamental. 2014;6: 493–506. doi:10.9789/2175-5361.2014v6n2p493

5. Carvalho e Silva Sales J, Ribeiro de AraÃ^o^jo MP, Cavalcante Coelho M, LÃ^o^cia Evangelista de Sousa Luz V, AraÃ^o^jo da Silva TC, JosÃ© Guedes da Silva JÃ^o^nior F. SEXUALITY OF PEOPLE LIVING WITH LEPROSY: PERCEPTION AND REPERCUSSIONS. Journal of Nursing UFPE / Revista de Enfermagem UFPE. 2013;7: 460–466. doi:10.5205/reuol.3073-24791-1-LE.0702201318

6. Chen IJ, Cheng SP, Sheu SJ. The meaning of physical activity for older adults with leprosy: A life story inside the wall. Leprosy Review. 2017;88: 399–409.

7. Correia JC, Golay A, Lachat S, Singh SB, Manandhar V, Jha N, et al. “If you will counsel properly with love, they will listen”: A qualitative analysis of leprosy affected patients’ educational needs and caregiver perceptions in Nepal. PLoS One. 2019;14. doi:10.1371/journal.pone.0210955

8. da Silva Duarte LMCP, Albino Simpson C, dos Santos Silva TM, de Lima Moura IB, Ramos Isoldi DM. SELF-CARE ACTIONS OF PEOPLE WITH LEPROSY. Journal of Nursing UFPE / Revista de Enfermagem UFPE. 2014;8: 2816–2822. doi:10.5205/reuol.6081-52328-1-SM.0808201432

9. da Silva Santos K, Magali Fortuna C, Fagundes Carvalho Gonçalves M, Matumoto S, Ribeiro Santana F, Marciano FM. Meaning of leprosy for people who have experienced treatment during the sulfonic and multidrug therapy periods. Revista Latino-Americana de Enfermagem (RLAE). 2015;23: 620–627. doi:10.1590/0104-1169.0323.2596

10. Da Silva MCD, Paz EPA. Experiences of people affected by leprosy in the health services: A hermeneutic approach. Leprosy Review. 2019;90: 172–182.

11. Dadun null;Peters Ruth;Lusli Mimi;Miranda-Galarza Beatriz;van Brakel Wim;Zweekhorst Marjolein;Damayanti Rita;Irwanto null;Bunders Joske; Exploring the Complexities of Leprosy-related Stigma and the Potential of a Socio-economic Intervention in a Public Health Context in Indonesia. 2016;27: 23.

12. Dako-Gyeke M, Asampong E, Oduro R. Stigmatisation and discrimination: Experiences of people affected by leprosy in Southern Ghana. Lepr Rev. 2017;88: 58–74.

13. Ebenso B, Ayuba M. “Money is the vehicle of interaction”: Insight into social integration of people affected by leprosy in Northern Nigeria. Leprosy Review. 2010;81: 99–110.

14. Ebenso B.;Newell J.;Emmel N.;Adeyemi G.;Ola B.; Changing stigmatisation of leprosy: an exploratory, qualitative life course study in Western Nigeria. 2019;4.

15. Gonçalves M, Prado M, Silva SSD, Santos KDS, Araujo PN, Fortuna CM. Work and Leprosy: women in their pains, struggles and toils. Rev Bras Enferm. 2018;71: 660–667. doi:10.1590/0034-7167-2017-0598

16. Heijnders ML. The dynamics of stigma in leprosy. Int J Lepr Other Mycobact Dis. 2004;72: 437–47. doi:10.1489/1544-581x(2004)72<437:Tdosil>2.0.Co;2

17. Jatimi Atika;Yusuf Ah;Andayani Sestu Retno Dwi; Leprosy Resilience with Disabilities Due to Illness: A Qualitative Study. 2020;5: 106.

18. Jha K, Choudhary RK, Shrestha M, Sah A. An assessment of women’s empowerment in mixed self-help groups in dhanusha district of nepal. Leprosy Review. 2020;91: 155–172.

19. Jung HG, Yang YK. Disease experiences of female patients with Hansen’s disease residing in settlement in Korea. International Journal for Equity in Health. 2020;19: N.PAG-N.PAG.

20. Khanna D.;de Wildt G.;de Souza Duarte Filho L. A. M.;Bajaj M.;Lai J. F.;Gardiner E.;de Araújo Fonseca A. M. F.;Lindenmeyer A.;Rosa P. S.; Improving treatment outcomes for leprosy in Pernambuco, Brazil: a qualitative study exploring the experiences and perceptions of retreatment patients and their carers. 2021;21.

21. Lima MCV, Barbosa FR, Santos DCMD, Nascimento RD do, D’Azevedo SSP. Practices for self-care in Hansen’s disease: face, hands and feet. Rev Gaucha Enferm. 2018;39: e20180045. doi:10.1590/1983-1447.2018.20180045

22. Dadun D, Peters RMH, van Brakel WH, Bunders JGF, Irwanto I, Regeer BJ. Assessing the Impact of the Twin Track Socio-Economic Intervention on Reducing Leprosy-Related Stigma in Cirebon District, Indonesia. Int J Environ Res Public Health. 2019;16. doi:10.3390/ijerph16030349

23. Lusli M, Zweekhorst MBM, Miranda-Galarza B, Peters RMH, Cummings S, Seda FSSE, et al. Dealing with Stigma: Experiences of Persons Affected by Disabilities and Leprosy. BioMed Research International. 2015;2015: 1–9. doi:10.1155/2015/261329

24. Nasir A.;Yusuf A.;Listiawan M. Y.;Harianto S.;Nuruddin null;Huda N.; Adaptive strategy of women’s leprosy in indonesia psychic experience of women with leprosy in living a community life. 2020;11: 312.

25. Nations MK, Lira GV, Catrib AM. Stigma, deforming metaphors and patients’ moral experience of multibacillary leprosy in Sobral, Ceará State, Brazil. Cad Saude Publica. 2009/06/09 ed. 2009;25: 1215–24. doi:10.1590/s0102-311x2009000600004

26. Palmeira IP, Ferreira MD. “the Body I Was and the Body I Am”: Conceptions of Women with Alterations Caused by Leprosy. Texto & Contexto Enfermagem. 2012;21: 379–386. doi:10.1590/S0104-07072012000200016

27. Palmeira IP, Moura JN, Epifane SG, Ferreira AMR, Boulhosa MF. Hansen’s Disease Patients’ Perceptions on Their Altered Fundamental Human Needs: Indications for Self-Care. Revista De Pesquisa-Cuidado E Fundamental Online. 2020;12: 319–325. doi:10.9789/2175-5361.rpcfo.v12.7069

28. Pelizzari V, de Arruda GO, Marcon SS, Fernandes CAM. Perceptions of people with leprosy about disease and treatment. Rev Rene. 2016;17: 466–474. doi:10.15253/2175-6783.2016000400005

29. Peters RMH, Dadun, Lusli M, Miranda-Galarza B, Van Brakel WH, Zweekhorst MBM, et al. The meaning of leprosy and everyday experiences: An exploration in Cirebon, Indonesia. Journal of Tropical Medicine. 2013. doi:10.1155/2013/507034

30. Peters RMH, Zweekhorst MBM, van Brakel WH, Bunders JFG, Irwanto. ‘People like me don’t make things like that’: Participatory video as a method for reducing leprosy-related stigma. Global Public Health. 2016;11: 666–682.

31. Poestges H. Leprosy, the key to another kingdom. Lepr Rev. 2011/09/06 ed. 2011;82: 155–67.

32. Ramasamy S, Govindharaj P, Kumar A, Panneerselvam S. Disclosure of Disease among Women affected by Leprosy: A Qualitative Study. Disability, CBR & Inclusive Development. 2020;31: 64–78. doi:10.47985/dcidj.393

33. Schuller I, van Brakel WH, van der Vliet I, Beise K, Wardhani L, Silwana S, et al. The way women experience disabilities and especially disabilities related to leprosy in rural areas in south Sulawesi, Indonesia. Asia Pacific Disability Rehabilitation Journal. 2010;21: 60–70.

34. Shieh C.;Wang H. H.;Lin C. F.; From contagious to chronic: A life course experience with leprosy in Taiwanese women. 2006;77: 113.

35. Shyam-Sundar V.;De Wildt G.;Virmond M. C. L.;Kyte D.;Galan N.;Prado null;Chauhan A.; A qualitative study exploring the perceived impact of race on leprosy-affected persons’ experiences of diagnosis and treatment of leprosy in southeast Brazil. 2021;93: 13.

36. Sillo S, Lomax C, De Wildt G, Fonseca MD, Galan NGD, Prado RBR. A temporal and sociocultural exploration of the stigma experiences of leprosy patients in Brazil. Leprosy Review. 2016;87: 378–395.

37. Silva C. Leprosy as a Neglected Disease and Its Stigma in the Northeast of Brazil. Indian journal of leprosy. 2014;2014: 53.

38. Steremberg Pires D’Azevedo S, Nunes de Freitas E, do Nascimento LO, dos Santos DCM, Delmondes do Nascimento R. PERCEPTION OF PATIENTS WITH LEPRA ABOUT THE SELF-CARE GROUPS. Journal of Nursing UFPE / Revista de Enfermagem UFPE. 2018;12: 1633–1639. doi:10.5205/1981-8963-v12i6a230855p1633-1639-2018

39. RAHMAWATI I. The experiences of people affected by leprosy who participated in self-care groups in the community. Lepr Rev. 2017;88: 1–11.

40. Thompson L, Ioteba N, Chambers S. Leprosy in Kiribati: the lived experience. Leprosy Review. 2020;91: 353–366.

41. Try L. Gendered experiences: marriage and the stigma of leprosy. Asia Pacific Disability Rehabilitation Journal. 2006;17: 55–72.

42. van ’t Noordende A. T.;Aycheh M. W.;Schippers A.; The impact of leprosy, podoconiosis and lymphatic filariasis on family quality of life: A qualitative study in Northwest Ethiopia. 2020;14.

43. van ’t Noordende A. T.;van Brakel W. H.;Banstola N.;Dhakal K. P.; The Impact of Leprosy on Marital Relationships and Sexual Health among Married Women in Eastern Nepal. 2016;2016.

44. van Haaren MA, Reyme M, Lawrence M, Menke J, Kaptein AA. Illness perceptions of leprosy-cured individuals in Surinam with residual disfigurements - “I am cured, but still I am ill.” Chronic Illn. 2017;13: 117–127. doi:10.1177/1742395316657398

45. Van Netten WJ, Van Dorst MMAR, Waltz MM, Pandey BD, Aley D, Choudhary R, et al. Mental wellbeing among people affected by leprosy in the Terai region, Nepal. Leprosy Review. 2021;92: 59–74. doi:10.47276/lr.92.1.59

46. Van’T Noordende A. T.;Lisam S.;Ruthindartri P.;Sadiq A.;Singh V.;Arifin M.;van Brakel W. H.;Korfage I. J.; Leprosy perceptions and knowledge in endemic districts in india and indonesia: Differences and commonalities. 2021;15: 19.

47. Yusuf A. null;Aditya R. S. null;Yunitasari E. null;Aziz A. N. null;Solikhah F. K. null; Experience of persons affected by leprosy in facing psychosocial problems: A qualitative method. 2020;11.

48. Sottie CA, Darkey J. Living with stigma: Voices from the Cured Lepers’ village in Ghana. Soc Work Health Care. 2019;58: 151–165. doi:10.1080/00981389.2018.1526842

49. Calcraft JH. The effects of the stigma of leprosy on the income generation of leprosy affected people in the Terai area of south east Nepal. Asia Pacific Disability Rehabilitation Journal. 2006;17: 73–89.

Legend

List of Study Findings with Illustrations
