# Supplementary material for: Experiences of living with leprosy: A systematic review and qualitative evidence synthesis
Source: PLoS Negl Trop Dis. 2022 Oct 5;16(10):e0010761. doi: 10.1371/journal.pntd.0010761 (PMC9576094; doi:10.1371/journal.pntd.0010761)
Supplement: S7 Appendix — (DOCX) [file pntd.0010761.s007.docx]

S7 Appendix

Summary of findings

| **Synthesised Findings** | **Type of research** | **Dependability** | **Credibility** | **ConQual Score** | **Comments** |
| --- | --- | --- | --- | --- | --- |
| **Biophysical Impact** *The physical presentation of leprosy, diagnosis and treatment, assessment of impairments and disabilities, and rehabilitation. Leprosy affects the performance of activities of daily living, including sexual activities. Education and information will influence their level of involvement in self-care.* | Qualitative | Downgrade 1 level | Downgrade 1 level | Moderate | Dependability: Most primary studies scored 5 or 6 out of 7. Dependability concerns were that studies had no statement locating the researcher culturally/theoretically [1,2] and no acknowledgment of their influence on the research ( [2,3].  Credibility: Downgraded one level due to a mix of unsupported and credible findings. N2, C39 |
| **Mental and Emotional Impact** *The understanding/knowledge of leprosy, including the myths/beliefs with regards to causation and transmission of the disease and its impact on their feelings and interactions with the people around them. Leprosy affects the person's mental, emotional, social, and spiritual well-being.* | Qualitative | Downgrade 1 level | Downgrade 2 level | Low | Dependability: Most primary studies scored 5 or 6 out of 7. Dependability concerns were that studies had no statement locating the researcher culturally/theoretically [1,2] and no acknowledgment of their influence on the research [2,3].  Credibility: Downgraded two levels due to a mix of unsupported and credible findings. N10, C35 |
| **Social Impact** *The interactions of individuals with leprosy and the challenges faced by women with their families and community, including institutionalisation and their coping strategies. Many of them draw on their religion and spiritual beliefs to support themselves.* | Qualitative | Downgrade 1 level | Downgrade 1 level | Moderate | Dependability: Most primary studies scored 5 or 6 out of 7. Dependability concerns were that studies had no statement locating the researcher culturally/theoretically [1,2]) and no acknowledgment of their influence on the research [2,3].  Credibility: Downgraded one level due to a mix of unsupported and credible findings. N6, C64 |
| **Economic Impact** *The employability of individuals with leprosy and the adaptations at work, skills development, financial assistance, and the economic impact on their families and community.* | Qualitative | Downgrade 1 level | Downgrade 1 level | Moderate | Dependability: Most primary studies scored 5 or 6 out of 7. Dependability concerns were that studies had no statement locating the researcher culturally/theoretically [1,2] and no acknowledgment of their influence on the research [2,3].  Credibility: Downgraded one level due to a mix of unsupported and credible findings. N1, C13 |

N – unsupported findings. C – Credible findings.

Dependability was assessed using Q 1 – 7 of the JBI Critical Appraisal framework (Appendix IV), and as most of the findings from the included studies had contributions to the four synthesised findings, the dependability assessment was generalised for all four synthesised findings.

Credibility was assessed using the ratio of unsupported findings to that of the credible findings from the extracted findings generated in the meta-aggregative flow chart of JBI Sumari software. (This was modified from the original paper by Munn et al. as we did not find any findings that would have been categorised as unequivocal.) [4]) (Appendix VII)

| **Ratio of Unsupported Findings (N)** | **Downgrade level** |
| --- | --- |
| 0-5% | No downgrade |
| 6-10% | 1 level downgrade |
| 11-30% | 2 level downgrade |
| 31-50% | 3 level downgrade |
| >50% | 4 level downgrade |

**Legend**

Summary of findings

**References**

1. Rai SS, Peters RMH, Syurina EV, Irwanto I, Naniche D, Zweekhorst MBM. Intersectionality and health-related stigma: insights from experiences of people living with stigmatized health conditions in Indonesia. International Journal for Equity in Health. 2020;19: N.PAG-N.PAG.
2. Chen IJ, Cheng SP, Sheu SJ. The meaning of physical activity for older adults with leprosy: A life story inside the wall. Leprosy Review. 2017;88: 399–409.
3. Araújo de Souza I, Aparecido Ayres J, Meneguin S, Spagnolo RS. Hansen’s disease patients’ perception of self-care from the complexity perspective. Anna Nery School Journal of Nursing / Escola Anna Nery Revista de Enfermagem. 2014;18: 510–514. doi:10.5935/1414-8145.20140072
4. Munn Z, Porritt K, Lockwood C, Aromataris E, Pearson A. Establishing confidence in the output of qualitative research synthesis: the ConQual approach. BMC Medical Research Methodology. 2014;14: 108. doi:10.1186/1471-2288-14-108
