# Supplementary material for: Experiences of living with leprosy: A systematic review and qualitative evidence synthesis
Source: PLoS Negl Trop Dis. 2022 Oct 5;16(10):e0010761. doi: 10.1371/journal.pntd.0010761 (PMC9576094; doi:10.1371/journal.pntd.0010761)
Supplement: Attachment — Submitted filename: PLOS NTD Leprosy review resubmission letter 30 July 2022.docx [file pntd.0010761.s008.docx]

30 July 2022

Manuscript Reference: PNTD-D-22-00695

Alberto Novaes Ramos Jr
Associate Editor
PLOS Neglected Tropical Diseases

Guilherme Werneck
Deputy Editor
PLOS Neglected Tropical Diseases

Dear Editors

Re: "Experiences of living with leprosy: A systematic review and qualitative evidence synthesis"

Thank you very much for your reviewers’ very kind and encouraging feedback. Their insightful and meaningful comments have been incorporated into the revised version which we are submitting here. These have indeed enhanced this review and we are grateful for the time and care afforded by the reviewers to this paper.

The following table summarises the relevant reviewers’ comments and our responses.

We look forward to your favourable response.

Yours sincerely

Dr Norana Abdul Rahman

Corresponding Author

[drnorana@gmail.com](mailto:drnorana@gmail.com)

norana.rahman@perdanauniversity.edu.my

| **Reviewers’ comments** | **Response** | **Revision in manuscript** | **Location** |
| --- | --- | --- | --- |
| **Methods**  Reviewer #1: Methods were clearly articulated.  Details are described in a way that others could use the same methodology in future studies and reviews.  It is unfortunate that articles had to be excluded due to language. | Thank you for your comments. The reason for the exclusion of non-English articles without full translation into English was that there were limited resources available for translational services and furthermore there were enough articles available for review. | Non-English articles without full translation into English were excluded due to limited resources available for translational services.  Non-English articles without full translation into English were also not included because of limited resources available for comprehensive translational services. | Lines 182-183  Lines 874 -876 |
| **Conclusions**  Reviewer #1: It was surprising to see that few studies were included from India in the analysis. As India is one of the countries with the greatest number of leprosy affected persons, the authors may want to comment why so few studies from India were in analysis and make a recommendations. | Thank you for your comments. The methodology applied in this review followed the strict search strategy and keywords used during the execution of the search of the electronic databases.  These words were constructed based on the inclusion and exclusion criteria. One of the reasons for the absence of any articles would be the absence of keywords within the title and abstract. Failure to include them will result in the search engines’ inability to detect and retrieve these articles. For transparency, quality and reproducibility only articles that fulfil the search strategy were made available for full analysis. To ensure that all future articles are included in future reviews it is important for authors to craft and develop abstracts and keywords to include all the keywords described in this review. | The search included keywords and free text terms for (synonyms of) 'Leprosy' combined with (synonyms of) 'quality of life' combined with (synonyms of) 'qualitative research'. The search strategy was also performed with the descriptors from the international vocabulary used in the health area, MeSH - Medical Subject Headings created by the National Library of Medicine for literature indexed on MEDLINE, combined through Boolean operators. Animal and child studies were excluded from the search. Studies published in any foreign language with English translation were included in the search. Non-English articles without full translation into English were excluded due to limited resources available for translational services.  Future researchers should ensure the inclusion of keywords in their titles and abstracts of their manuscripts to ensure visibility by search engines. | Lines 176 – 183  Lines 926-927 |
| **Conclusions**  Reviewer #1: Check Figure 2: The effects of leprosy on the lives of leprosy-affected people helped the reader visualize the Impact of each category along with an illustration.  After reading I was surprised that the Biomedical effects(2/10) of leprosy and functional impairments and disability3/10) did not have a line drawn to mental and emotional impact.  See lines 759-763.  Also, the Social effects of leprosy (9/10) did not have a line to draw to Biophysical Impact.  See lines 774-776 which talk about treatment adherence. | Thank you for your comments we agree with your suggestions and have incorporated them into our revised manuscript. | Figure has been amended accordingly. | Line 857, Page 58 |
| **Editorial and Data Presentation Modifications**  Reviewer #1: Check Figure 2 for  accuracy based on my comments above.  Reviewer #3:  Since the epidemiological record containing data for the year 2020, released in September 2021, is now available, I advise amending reference number nine  In the results session, topic 3.1, line 259, it is described that 361 articles were excluded. Authors should make it clear to the reader the reason for exclusion at this stage. Also, add this data to the PRISMA diagram (figure 1).  On line 376, check the appendix number. According to the data presented, I believe that appendix VI should be written.  On line 403, check the reference made to the illustration in question. I did not find the illustration with identification #2 in the appendix, in the part that refers to reference study 58. | Thank you. We have incorporated your suggestions as advised.  Thank you for your comments. We have amended reference number 9 to the latest epidemiological record September 2021  Thank you. We have incorporated your suggestions and added the same to the PRISMA diagram.  Thank you for your observation. We have changed it accordingly.  Thank you for the observation and we have corrected the omitted entry of #2. | Figure has been amended accordingly.  The global prevalence of leprosy at the end of 2020 was 129,192, with a corresponding prevalence rate of 16.6 per million population. (9) There were 127,396 new cases detected worldwide, with a new case detection rate of 16.4 per million population, and these figures were much lower than in previous years, probably due to reduced leprosy prevention activities caused by the COVID-19 pandemic resulting in less detection and reporting of cases. (9)  A total of 446 titles and abstracts were screened for inclusion. A further 361 articles were removed because they did not fulfil the selection criteria,  Records excluded because titles and abstracts did not match the inclusion criteria (n = 361)  Appendix VI shows the complete list of study findings and their illustrations.  Back then, I had eyebrows, hands, and feet... People didn’t recognize me because my face became  swollen and my appearance changed. (#2) | Line 857, Page 58  Lines 86 – 91  Lines 264 – 265  Lines 291 - 292  Lines 382  Please Refer Appendix VI Page 42 |
| **Summary and General Comments**  Reviewer #1  The greatest limitation was the study had few qualitative studies from India and could only review English studies. This work could guide the future study of qualitative data in non-English studies as well as English. It would be interesting to see if the non-English studies not included in this analysis would find similar or different categories. | We agree that the inclusion of non-English articles should be a subject of future reviews and may shed further light on the categories. | Non-English articles should be considered in future reviews and may shed further light on the subject. | Lines 927 -928 |
